# Network analysis of functional disabilities and their association with mental well-being in children and adolescents: multi-country study across low- and middle-income countries

**DOI:** 10.1192/bjp.2024.278

**Published:** 2026-02

**Authors:** Shanquan Chen, Emilio Fernandez-Egea, Sara Rotenberg, Rudolf N. Cardinal, Daiane Borges Machado, Tracey Smythe, Tamsin J. Ford, Hannah Kuper

**Affiliations:** 1 International Centre for Evidence in Disability, London School of Hygiene & Tropical Medicine, London, UK; 2 Cambridgeshire and Peterborough NHS Foundation Trust, Cambridge, UK; 3 Department of Psychiatry, University of Cambridge, Cambridge, UK; 4 Behavioural and Clinical Neuroscience Institute, University of Cambridge, Cambridge, UK; 5 Department Global Health and Social Medicine, Harvard Medical School, Boston, Massachusetts, USA; 6 Center of Data and Knowledge Integration for Health (CIDACS), Fiocruz, Salvador, Brazil; 7 Division of Physiotherapy, Department of Health and Rehabilitation Sciences, Stellenbosch University, Cape Town, South Africa

**Keywords:** Anxiety, children and adolescents, depression, functional disabilities, mental well-being

## Abstract

**Background:**

To develop effective mental health interventions for children and adolescents, it is essential to understand the intricate link between functional disability and mental well-being in this group.

**Aims:**

To explore the network connections between various aspects of functional disability and mental well-being in young people with disabilities.

**Method:**

We analysed data from the Multiple Indicator Cluster Surveys in 47 low- and middle-income countries, tracking progress towards health-related sustainable development goals. Our focus was on children and adolescents aged 5–17 with functional disabilities. Mental well-being was gauged using carer-reported signs of depression, anxiety and disability on the Child Functioning Module. Network-analysis techniques were used to examine links between mental well-being and functional disability domains.

**Results:**

The study included 32 669 eligible children aged 5–17 with functional disabilities (14 826 females and 17 843 males). The core domains of disability with the strongest connections to poor mental well-being were difficulties in accepting change, making friends, behavioural control (controlling own behaviour) and remembering/concentrating. These associations remained largely consistent across different genders and developmental stages. However, there were notable gender differences and age-related shifts in the relationships between specific disabilities and mental well-being. In particular, signs of anxiety in males and depression in females were most associated with functional disability overall, while signs of depression had the closest links to disability in adolescents.

**Conclusions:**

The network perspective may enable the design of tailored interventions and support services that consider age and gender differences. Further research should continue to explore these complex relationships, incorporating novel methodologies like network-analysis to enhance the understanding of these associations.

Globally, approximately 207.4 million children aged 5–17 years experience moderate-to-severe disabilities.^1^ These children often face increased risks of mental health conditions such as depression and anxiety in comparison with their non-disabled peers.^2–5^ One reason is that this developmental stage is characterised by comparisons with peers and the development of self-esteem, which can be adversely affected by perceived differences such as limited mobility.^6^ The implications of these risks during childhood are great. Research has demonstrated that childhood mental health problems are associated with adverse outcomes later in life, including compromised development, social and emotional well-being, poor quality of life and even suicide.^7,8^ Therefore, effective management of mental health in children with functional disabilities is essential to promoting long-term positive outcomes and well-being.

Existing literature examining the association between functional disability and mental illness has typically treated disability as a dichotomous variable or focused on specific difficulties (e.g. communication,^3^ concentration^5^ or learning^2^). One study was identified that investigated the association between the number of types of disability and mental health problems.^4^ However, to the best of our knowledge, no study has specifically addressed the fact that multiple functional disabilities (such as hearing and communication difficulties) may coexist within the same person. Furthermore, these coexisting multi-functional disabilities frequently exhibit reciprocal associations, exacerbating one another and influencing mental health outcomes.^4^ For instance, a communication disability can affect social interactions and self-esteem, which, in turn, may contribute to increased mental health difficulties such as anxiety or depression.^3^ Similarly, mental health conditions can affect communication abilities, making it more challenging to express oneself effectively.^3^ This knowledge gap poses significant challenges in developing effective interventions to improve the mental health of children with functional disabilities.

## Network analysis

Network analysis is an emerging statistical method in psychiatric research that has the potential to address the challenges mentioned above. In a network, nodes represent elements (e.g. symptoms of disorders, biological components and environmental risk factors) and edges signify the relationships between nodes.^9^ Network analysis enables the quantification of centrality indices, which indicate the importance of a node in terms of its connectivity and putative role in maintaining the network.^9^ The network theory of psychopathologies assumes an interplay or interconnection between nodes, where symptoms are conceptualised as distinct entities that can influence one another and also reinforce each other within the network.^10^ The premise that symptoms are interconnected and can mutually influence each other within the network underscores the potential advantages of employing network analysis to effectively address the collinearity among variables of interest. Arguably, network analysis is especially useful when investigating links between different domains of interest, such as comorbid features, risk factors and symptomatology, as it can identify specific bridging components (e.g. nodes, links) that may serve as effective intervention targets.^11^

## The current study

In this study, we employed network analysis to examine associations between functional disability and mental well-being in children and adolescents in low- and middle-income countries (LMICs), conceptualising functional domains as components of overall disability. The analysis incorporated gender-specific comparisons, given documented male–female differences in disability and mental health, to inform gender-sensitive interventions. Age-specific analysis (children 5–9 years old versus adolescents 10–17 years old) identified developmental patterns in disability–mental illness associations. Research questions addressed were: (a) domains of functional disabilities connected with mental well-being; (b) gender differences in these connections; and (c) consistency of relationships across developmental stages.

## Method

### Data sources and study sample

We used data from the sixth round of the Multiple Indicator Cluster Surveys (MICS), collected between 2016 and 2021 in LMICs. The MICS were initiated in the mid-1990s and are the primary data source used by United Nations agencies to measure progress towards health-related sustainable development goals.^12^ The MICS followed a probabilistic, clustered, stratified and multistage sampling strategy to collect national representative data on children, adults and households.^12^ Detailed information about the MICS, including the questionnaire, sampling methods and quality control procedures, can be found elsewhere.^12,13^ The sixth round of MICS used a standardised survey (to make it comparable) that included indicators related to sociodemographic factors, functional disabilities and mental well-being. The MICS data comprised separate data-sets containing individual- and household-level information. For this study, we utilised and merged three specific data-sets: the children’s file containing data for ages 5–17 years (fs.sav), the household information file (hh.sav) and the household members file (hl.sav). These data-sets were linked using unique household identifiers and individual identification numbers as the merging variables.

The data are publicly available. The London School of Hygiene and Tropical Medicine research ethics committee approved this project (reference number 22719). Participants in the original studies gave informed consent and each study was approved by relevant institutional ethics review committees within each country involved in data collection.

### Participants

For this study, we focused on samples of children aged 5–17 years across 47 participating LMICs. Individuals aged 5–9 were defined as children and those aged 10–17 were defined as adolescents. To enable us to look at effects in young people who have disabilities, eligible individuals had functional disabilities, as described below. We excluded cases with missing values (3342 cases, 9.3%). Supplementary Fig. 1 (available at https://doi.org/10.1192/bjp.2024.278) shows the flowchart of selection of participants.

### Measures

#### Functional difficulty and mental well-being

Functional difficulty and mental well-being were measured by the Child Functioning Module (CFM), which has been validated for use in surveys with mothers or primary caregivers as proxy respondents.^4^ The CFM was developed by the United Nations Children’s Fund (UNICEF) and the Washington Group on Disability Statistics, based on the World Health Organization International Classification of Functioning, Disability and Health (ICF) and the biopsychosocial model of disability.^14^ The CFM has undergone extensive review by experts and testing in several countries to determine the quality of questions being asked and to ascertain cultural understanding by respondents who speak different languages or with different types or levels of disability.^14^ In 2017, a joint statement issued by multiple United Nations agencies, member states, organisations representing persons with disabilities and other stakeholders recommended the CFM as the appropriate tool for sustainable development goals (SDG) data disaggregation for children.^14^

The CFM for children aged 5–17 years has 24 questions and covers domains on vision/seeing, hearing, mobility/walking, self-care, communication/comprehension, learning, remembering, focusing attention/concentrating, coping with change, behavioural control (controlling own behaviour), relationships/making friends and mental well-being. The schedule is described in detail elsewhere.^13^ For the domains except for mental well-being, the presence and severity of functional difficulties were captured by a response scale covering four categories: ‘no difficulty’, ‘some difficulty’, ‘a lot of difficulty’ or ‘cannot do at all’, which were coded 1 to 4 respectively. Consequently, within this context, reference to a specific domain inherently implies increased challenges within that particular domain, unless explicitly stated otherwise. We summed the sub-questions for domains with more than one question and rescaled the scores to a consistent maximum of 4. Eligible participants, following UNICEF’s definition, were considered to have a functional disability if ‘a lot of difficulty’ or ‘cannot do at all’ were reported on at least one of the aforementioned domains.^4^

Mental well-being was captured by two questions on signs of depression (‘How often does the child seem very sad or depressed?’) and anxiety (‘How often does the child seem very anxious, nervous, or worried?’) by asking their parent or caregiver, with possible responses of ‘daily’ (coded 5), ‘weekly’ (coded 4), ‘monthly’ (coded 3), ‘a few times a year’ (coded 2) and ‘never’ (coded 1).

#### Covariates

We investigated the following sociodemographic characteristics: age, gender (male versus female), educational level (pre-primary or none, primary, and secondary or above) and socioeconomic status, which was measured by wealth index (available in the data directly and derived from a principal component analysis based on household assets, characteristics and infrastructure).^15^ We also investigated place of residence (rural versus urban),^4^ time involved in economic activity and time spent on house chores, because of the evidence of associations of these factors with disabilities or mental well-being.^16^ The question phrasing relating to these last two items can be found elsewhere.^13^

### Statistical analysis

#### Basic description

We reported categorical variables as numbers and percentages, and continuous variables as means and standard deviations (s.d.). Differences between gender were assessed via two-tailed *t*-tests for continuous variables and chi-squared tests for categorical variables (such as place of residence, educational level and socioeconomic status).

#### Network estimation

Networks consist of nodes (domains) and edges (connections or associations between nodes). In this study, the network consisted of a ‘community’ of mental well-being nodes (two nodes), a community of disabilities (ten nodes) and a community of confounders (six nodes). Thicker edges in the networks indicate stronger associations between nodes. Because scores for each node had skewed distributions, the network analysis in this study was conducted using the graphical least absolute shrinkage and selection operator (GLASSO) method, with the optimal degree of shrinkage selected based on the extended Bayesian information criterion (EBIC) under a default hyperparameter value (*γ* = 0.5).^9^ The GLASSO method estimates regularised partial correlations between nodes, controlling for the associations of all other nodes in the network.^9^ In this study, we fed the EBIC-GLASSO procedure with a correlation matrix, which was estimated based on available cases and considering the sampling weights. Sampling weights were calculated based on the non-response rate, accounting for selection stages and selection probabilities. The weight values were provided directly in the MICS data-sets. Details of how the weights were calculated can be found elsewhere.^12^

#### Centrality estimation

One-step bridge expected influence (BEI) was calculated to identify the bridge nodes between the community of mental well-being and the community of disabilities. One-step BEI is the sum of all signed values of the edges between a node and all the other nodes from a different community, and quantifies the total strength of the connections between that node and the other community. A higher BEI value indicates stronger connections between communities, suggesting that the node has greater potential influence on (or is more influenced by) nodes in the other community.

We also calculated a network centrality index, namely expected influence (EI), for additional testing. Expected influence was defined as the sum of all edges extending from a given node (where the sign of each edge is maintained). A higher EI value indicates that a node has stronger overall connections with all other nodes in the network, suggesting it may be more central or influential in the entire network structure. Although several centrality indices are commonly used in network analysis (e.g. degree, betweenness and closeness centrality), we selected the EI because it accounts for both positive and negative relationships between nodes and has shown greater stability in psychological networks.^17^ This makes it particularly suitable for examining the complex relationships between functional disabilities and mental well-being.

#### Network comparison

Networks for females and males were compared using the global strength invariance test, which tests the null hypothesis that the weighted absolute sum of all edges in the network is the same for both genders. A higher global strength indicates stronger overall connectivity within the network, suggesting more robust relationships between all variables. Additionally, the network structure invariance test was employed, which tests the null hypothesis that the matrices of all edges are identical for both genders.^18^ A higher structural invariance value indicates greater differences in how variables are connected to each other between the two networks, suggesting distinct patterns of relationships in males versus females. To evaluate the difference in the BEI between genders, a permutation test was used and corrected by Benjamini–Hochberg method.^19^ The same network comparison was conducted between children (aged 5–9) and adolescents (aged 10–17) to examine potential differences.

#### Robustness analysis

We tested the accuracy and stability of the network using 5000 non-parametric bootstrapping (resampling participants from the data with replacement).^9^ This procedure generates 95% CIs of edge and centrality values. Less overlapping of confidence intervals between nodes suggests greater accuracy of edge or centrality.^9^

To test the stability of centralities in the networks, we adopted 5000 case-dropping bootstrapping (dropping participants from the data),^9^ which calculates the correlation stability coefficients. The correlation stability coefficient quantifies the maximum proportion of the original sample that can be dropped while continuing to estimate centrality values that correlate highly (*r* > 0.7) with the network from the full sample. Values of 0.25 and 0.5 indicate benchmarks for adequate and good network stability respectively.^9^ Centralities with correlation stability coefficients ≥0.25 can be regarded as interpretable.^9^

We also did a sensitivity analysis by performing 10 multiple imputations by chained equations, incorporating all study variables, to avoid biases due to missing data.

Analyses were performed using R (version 4.2.2 for Windows). Statistical significance was defined as *P* < 0.05. All tests were two-tailed.

## Results

Among 409 306 children covered by the sixth-round MICS, 36 001 (8.8%) were identified as having functional difficulties (Supplementary Fig. 1). After excluding 3342 cases with missing values, a total of 32 669 eligible children aged 5–17 with functional disabilities were included in this study (14 826 females and 17 843 males). Table 1 summarises their basic characteristics. There were no gender differences in the basic characteristics, including age, place of residence, socioeconomic status, except that a higher proportion of males belonged to the primary educational level (*P* < 0.001), males spent significantly more time involved in economic activities (*P* < 0.001) and females spent significantly more time on house chores (*P* < 0.001). There were no gender differences in the prevalence of difficulties hearing, remembering/concentrating and anxiety (*P* > 0.05), but a higher proportion of females reported difficulties seeing (*P* < 0.001), walking (*P* < 0.001) and making friends (*P* < 0.001) and depression (*P* < 0.001), whereas a higher proportion of males reported difficulties with self-care (*P* = 0.010), communication (*P* < 0.001), learning (*P* < 0.001), accepting change (*P* < 0.001) and behavioural control (*P* < 0.001).

**Table 1:** Basic description of sociodemographic and functional disability of participants

	All (*n* = 32 669)	Female (*n* = 14 826)	Male (*n* = 17 843)	*P*^a^
Age, years: mean (s.d.)	9.91 (3.77)	9.87 (3.78)	9.94 (3.77)	0.113
Educational level, *n* (%)				
Pre-primary or none	10 836 (33.2)	5117 (34.5)	5719 (32.1)	<0.001
Primary	16 415 (50.2)	7264 (49.0)	9151 (51.3)	
Secondary or above	5418 (16.6)	2445 (16.5)	2973 (16.7)	
Place of residence (= urban), *n* (%)	11 327 (34.7)	5190 (35.0)	6137 (34.4)	0.252
Wealth status (decile), *n* (%)				
Poorest	4502 (13.8)	2036 (13.7)	2466 (13.8)	0.748
2	4167 (12.8)	1886 (12.7)	2281 (12.8)	
3	3749 (11.5)	1713 (11.6)	2036 (11.4)	
4	3495 (10.7)	1526 (10.3)	1969 (11.0)	
5	3291 (10.1)	1494 (10.1)	1797 (10.1)	
6	3147 (9.6)	1434 (9.7)	1713 (9.6)	
7	2939 (9.0)	1359 (9.2)	1580 (8.9)	
8	2809 (8.6)	1289 (8.7)	1520 (8.5)	
9	2393 (7.3)	1088 (7.3)	1305 (7.3)	
Richest	2177 (6.7)	1001 (6.8)	1176 (6.6)	
Time involved in economic activity, hours per week: mean (s.d.)	2.41 (8.53)	1.76 (6.93)	2.95 (9.62)	<0.001
Time spent on house chores, hours per week: mean (s.d.)	5.21 (11.30)	6.84 (13.14)	3.85 (9.30)	<0.001
Functional disability (higher means more disability): mean (s.d.)				
Seeing	1.23 (0.59)	1.24 (0.60)	1.21 (0.57)	<0.001
Hearing	1.16 (0.50)	1.16 (0.51)	1.15 (0.50)	0.216
Walking	1.52 (0.80)	1.56 (0.81)	1.49 (0.79)	<0.001
Self-care	1.34 (0.73)	1.33 (0.72)	1.35 (0.74)	0.010
Communication	1.27 (0.62)	1.26 (0.61)	1.28 (0.63)	<0.001
Learning	1.62 (0.84)	1.59 (0.82)	1.64 (0.85)	<0.001
Remembering/concentrating	1.62 (0.82)	1.62 (0.82)	1.62 (0.82)	0.570
Accepting change	1.82 (0.90)	1.79 (0.89)	1.85 (0.90)	<0.001
Behavioural control	1.96 (0.93)	1.87 (0.91)	2.04 (0.94)	<0.001
Making friends	1.42 (0.80)	1.45 (0.82)	1.40 (0.78)	<0.001
Mental well-being (higher means more depression/anxiety)				
Depression	1.26 (1.36)	1.29 (1.36)	1.24 (1.35)	0.001
Anxiety	1.44 (1.44)	1.43 (1.43)	1.45 (1.46)	0.379

a .*P*-values for educational level, place of residence and socioeconomic status were obtained by chi-squared test, and for other variables via *t*-test.

**Fig. 1:**
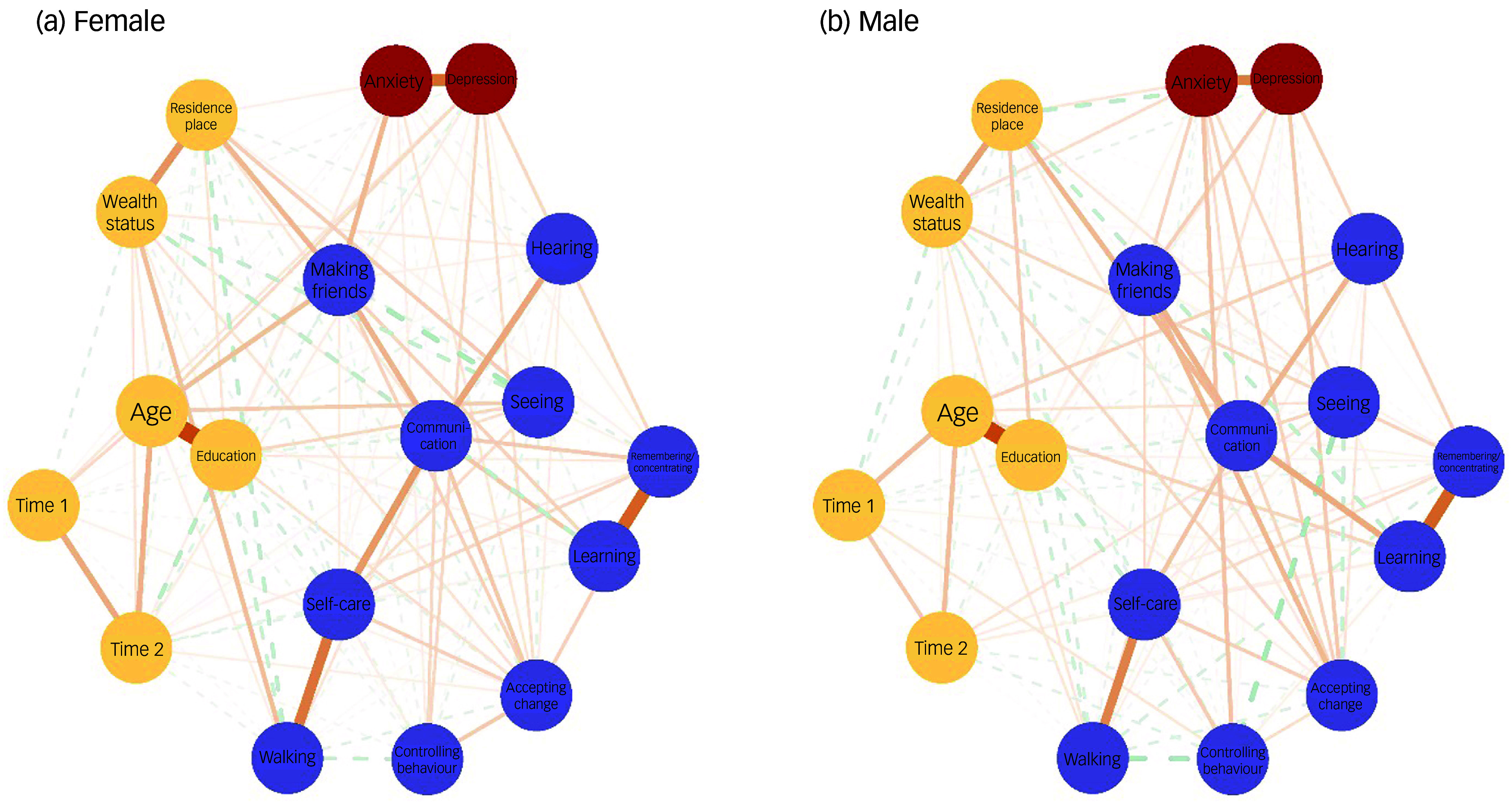
Network structure between functional disability and mental well-being, by gender. The nodes with different colours represent the ‘communities’ of mental well-being, disabilities and confounders. Edges represent the connections or associations between nodes, with thicker edges indicating stronger associations. A solid edge means a positive association and a dashed edge means a negative association. Reference to a specific domain (e.g. ‘Depression’, ‘Seeing’) implies increased difficulties within that particular domain.

**Fig. 2:**
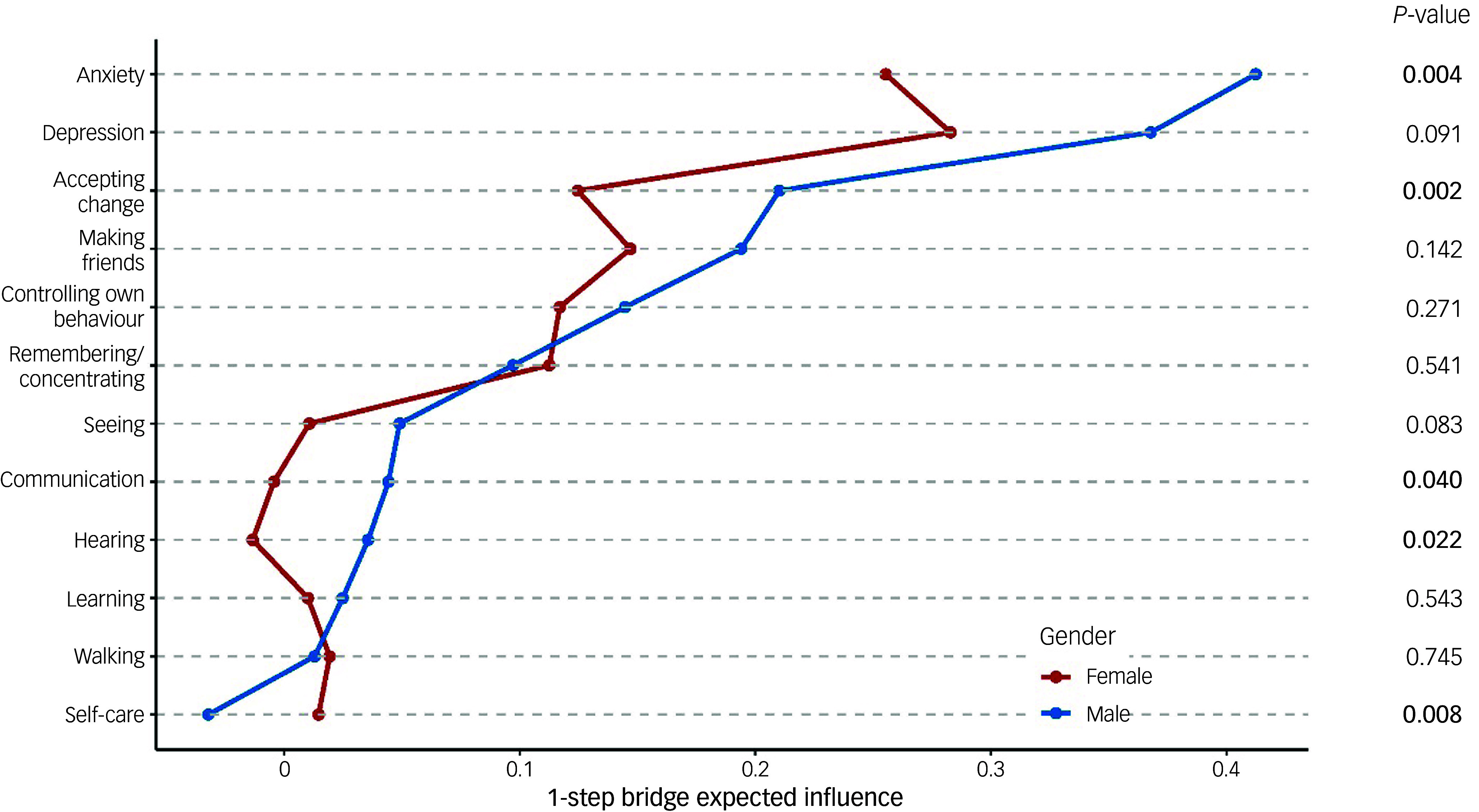
Bridge expected influence of nodes for functional disability and mental well-being, by gender. A node with a higher bridge expected influence serves as a more important link between the ‘community’ of functional disability nodes and the community of mental well-being nodes. *P*-values, comparing the two conditions for each node, were extracted from the permutation test and corrected by Benjamini–Hochberg method. Reference to a specific domain (e.g. ‘Depression’, ‘Seeing’) implies increased difficulties within that particular domain.

Figure 1 displays the separate networks for females and males. Statistical comparisons of networks between genders revealed a significantly higher global strength (0.608, *P* = 0.0176) and structural invariance (0.192, *P* < 0.001) in males than in females. Figure 2 provides a detailed examination of gender differences in BEI between the communities of mental well-being and functional disability. Within mental well-being, anxiety was the strongest factor connected with functional disability in males, whereas depression held this position for females (Fig. 2). Within functional disability, the top four nodes exhibiting the strongest connections to mental well-being were consistent across both genders, albeit with slight differences in their order. For males, the top four nodes were accepting change, making friends, behavioural control and remembering/concentrating, whereas for females, they were making friends, accepting change, behavioural control and remembering/concentrating (Fig. 2). Compared with females, males demonstrated significantly stronger BEI in anxiety (*P* = 0.004), accepting change (*P* = 0.002), communicating (*P* = 0.040) and hearing (*P* = 0.022), but exhibited significantly weaker BEI in self-caring (*P* = 0.008) (Fig. 2).

**Fig. 3:**
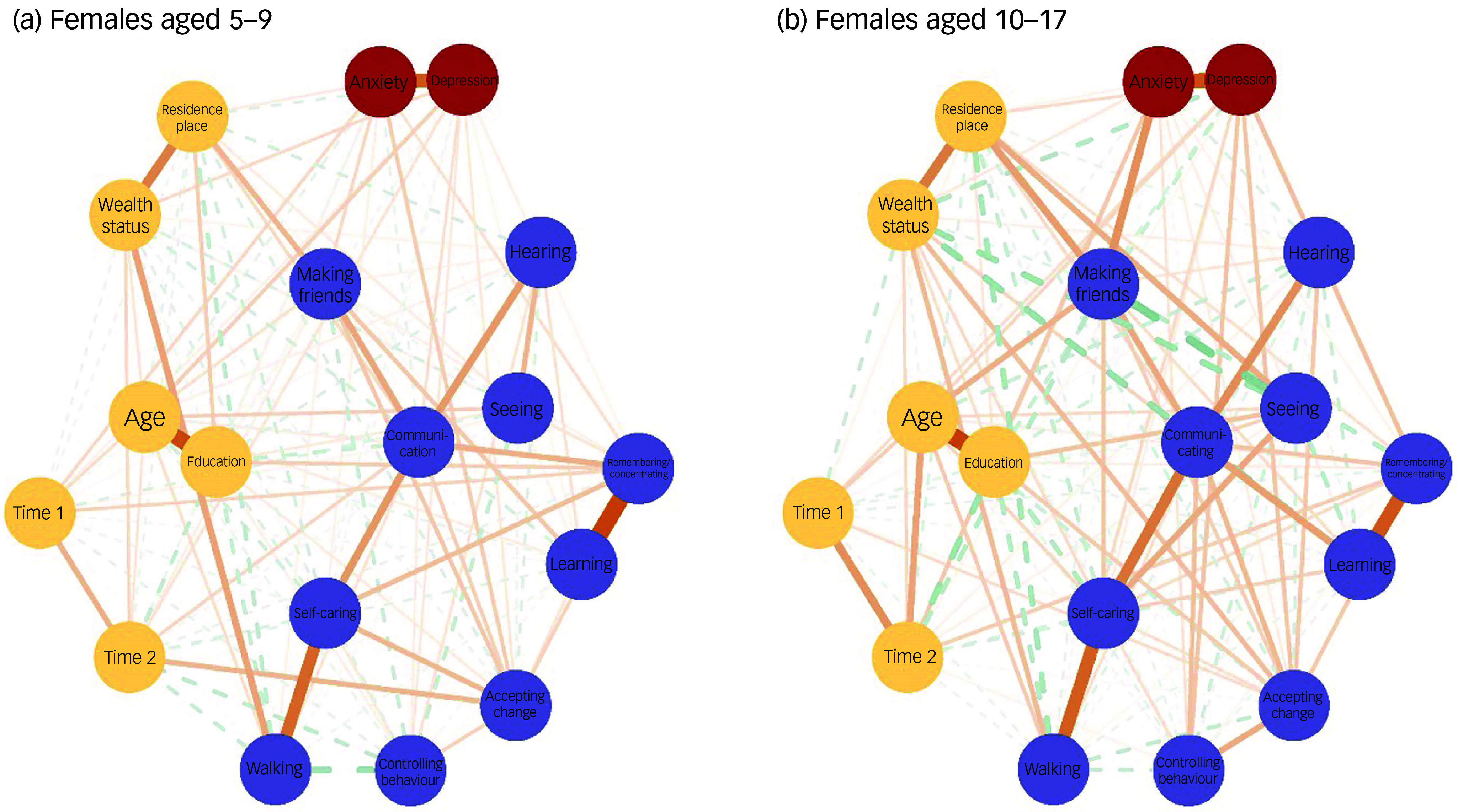
Network structure between functional disability and mental well-being among females, by age. The nodes with different colours represent the ‘communities’ of mental well-being, disabilities and confounders. Edges represent the connections or associations between nodes, with thicker edges indicating stronger associations. A solid edge means a positive association and a dashed edge means a negative association. Reference to a specific domain (e.g. ‘Depression’, ‘Seeing’) implies increased difficulties within that particular domain.

**Fig. 4:**
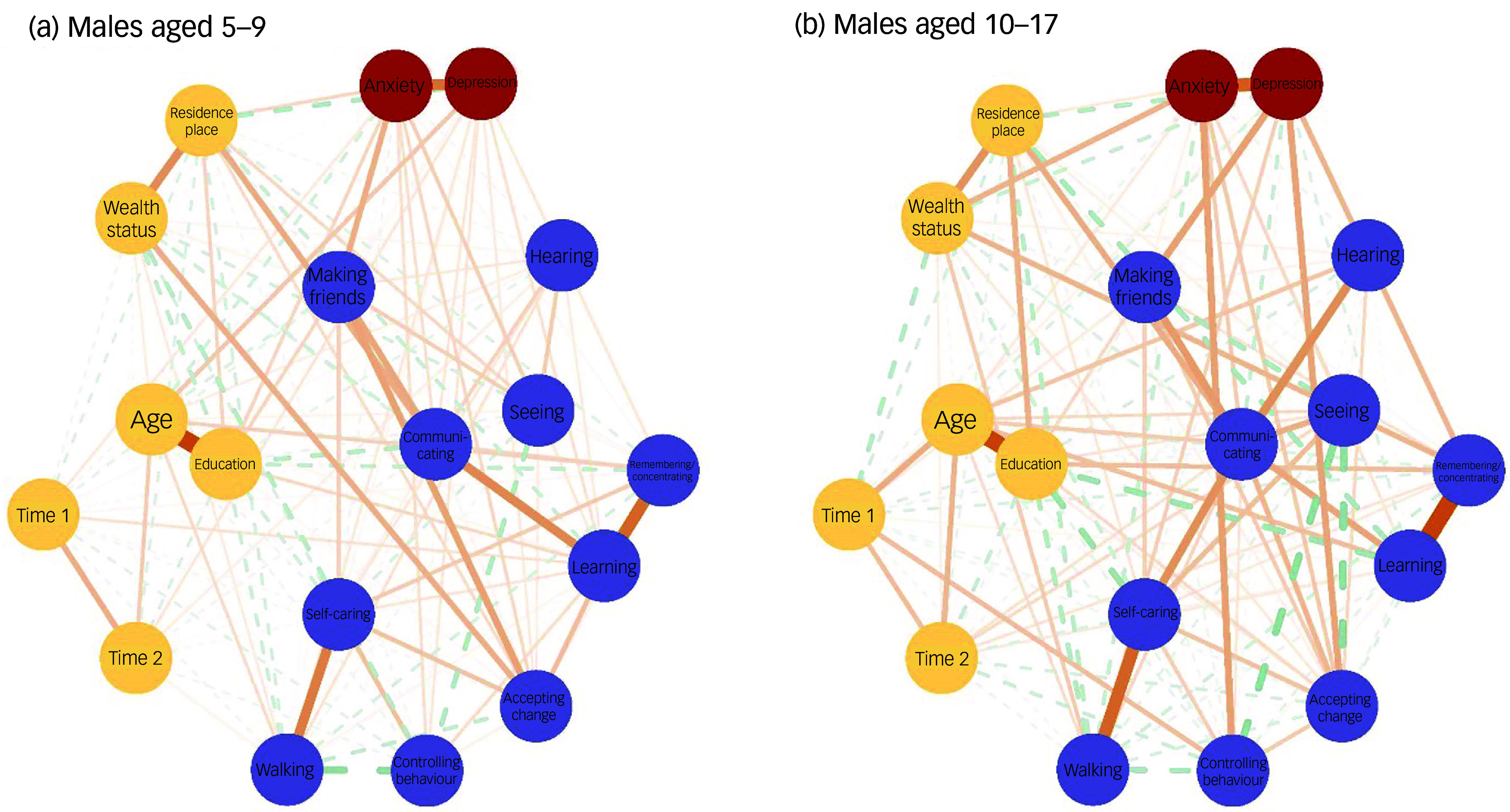
Network structure between functional disability and mental well-being among males, by age. The nodes with different colours represent the ‘communities’ of mental well-being, disabilities and confounders. Edges represent the connections or associations between nodes, with thicker edges indicating stronger associations. A solid edge means a positive association and a dashed edge means a negative association. Reference to a specific domain (e.g. ‘Depression’, ‘Seeing’) implies increased difficulties within that particular domain.

**Fig. 5:**
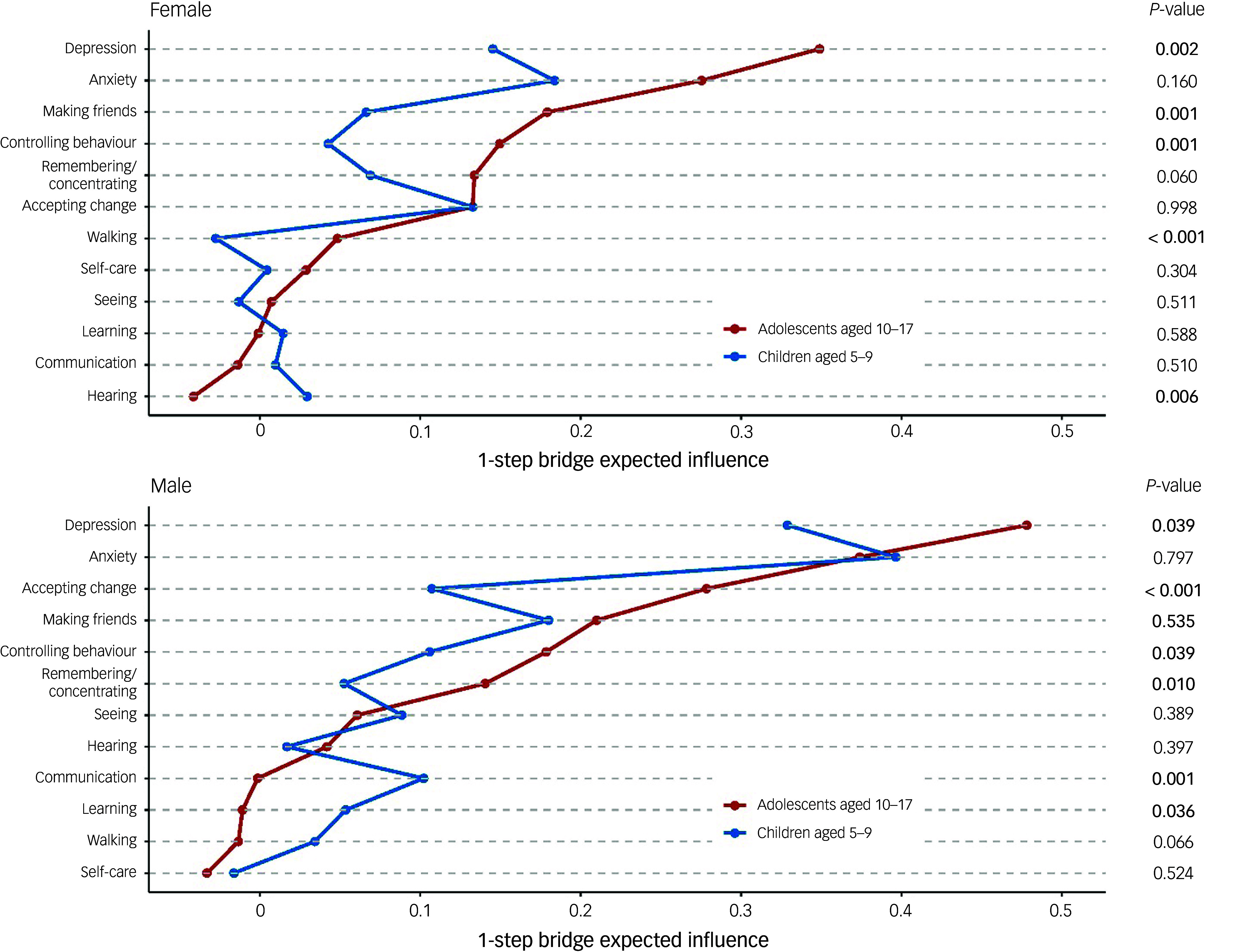
Bridge expected influence of nodes for functional disability and mental well-being, by gender and by age. A node with a higher bridge expected influence serves as a more important link between the ‘community’ of functional disability nodes and the community of mental well-being nodes. *P*-values, comparing the two conditions for each node, were extracted from the permutation test and corrected by Benjamini–Hochberg method. Reference to a specific domain (e.g. ‘Depression’, ‘Seeing’) implies increased difficulties within that particular domain. Factors sorted within each gender by the bridge expected influence for adolescents.

Figures 3 and 4 depict the networks for children and adolescents separately, for females and males respectively. The networks for both females and males exhibited significantly higher global strength (females: 2.987, *P* < 0.001; males: 2.305, *P* < 0.001) and structural invariance (females: 0.227, *P* < 0.001; males: 0.251, *P* < 0.001) in adolescents (10–17 years old) than in children (5–9 years old). Figure 5 presents a detailed comparison of children versus adolescents regarding the BEI between mental well-being and functional disability for females and males separately. Among mental well-being nodes, anxiety had the strongest connection with functional disability for children (5–9 years old), whereas depression was the strongest for adolescents (10–17 years old), a pattern observed in both females and males (Fig. 5). Within functional disability, the top four nodes with the strongest connections to mental well-being were similar for children and adolescents but varied in their order, with nearly inverted rankings observed for females versus males. For the younger females, the top four nodes (in descending order) were accepting change, remembering/concentrating, making friends and behavioural control, whereas for adolescent females, they were making friends, behavioural control, remembering/concentrating and accepting change (Fig. 5(a)). For younger males, the top four nodes were making friends, accepting change, behavioural control and communication, whereas for adolescent males, they were accepting change, making friends, behavioural control and remembering/concentrating (Fig. 5(b)). Compared with younger females, adolescent females exhibited significantly stronger BEI for depression (*P* = 0.002), making friends (*P* = 0.001), behavioural control (*P* = 0.001) and walking (*P* < 0.001), but demonstrated significantly weaker BEI for hearing (*P* = 0.006) (Fig. 5(a)). In contrast, compared with younger males, adolescent males had significantly stronger BEI for depression (*P* = 0.039), accepting change (*P* < 0.001), behavioural control (*P* = 0.039) and remembering/concentrating (*P* = 0.010), but showed significantly weaker BEI for communicating (*P* = 0.001) and learning (*P* = 0.036) (Fig. 5(b)).

Supplementary Figs 2–4 present the EI of each node in the networks depicted in Figs 1, 3 and 4, highlighting that the EI of certain functional disability nodes (e.g. communication) is greater than that of mental well-being nodes. The discrepancy between EI and BEI suggests that these types of disability may have a more substantial impact within the domain of disability itself rather than a bridge impact on mental well-being.

The robustness analysis employing 5000 bootstraps (Supplementary Figs 5–10) confirmed the stability of our findings. In general, the bootstrapped 95% confidence intervals for the estimated edge weights and centrality measures were narrow, indicating the reliability of the network estimates (Supplementary Figs 11–16). The correlation stability coefficients for BEI were 0.739 for females, 0.475 for child females, 0.633 for adolescent females, 0.844 for males, 0.633 for child males and 0.739 for adolescent males. All the correlation stability coefficients were either above or in close proximity to the preferred 0.5 cut-off, suggesting that even with a 50% reduction in the corresponding participants randomly, the stability of the respective networks would be maintained.

The sensitivity analysis, which employed multiple imputation to handle missing data (Supplementary Figs 17–21), corroborated the consistency of the primary findings, including the gender- and age-specific disparities between the connection of functional disabilities and mental well-being, as well as the core domains of disability that have the strongest connections to poor mental well-being.

## Discussion

### Principal findings

For what we believe to be the first time, the current study adopted a network analysis perspective to explore the relationship between domains of disability and mental well-being among children and adolescents with disabilities, as well as the role of disability in the presence of anxiety and depression from childhood to adolescence. Our network analysis revealed three key findings: (a) the mental well-being of males was more closely connected with functional disability than that of females; (b) for both genders, the network between functional disability and mental well-being transformed from childhood to adolescence, with depression emerging as the symptom that exhibited the strongest connection to functional disability, surpassing anxiety; and (c) the disability domains with the strongest connections to poor mental well-being remained largely consistent, encompassing accepting change, making friends, behavioural control and remembering/concentrating. It is noteworthy that the order and the strength of the connections varied across both genders and developmental stages.

The observed gender differences in associations between mental well-being and functional disability corroborate existing literature,^20–23^ while extending it through network analysis that reveals distinct patterns in associations of symptoms of depression and anxiety with various disabilities. These differences may be attributed to gender-specific development of cognitive and emotion regulation strategies,^24^ alongside differential coping mechanisms.^22,23^ Males typically employ problem-focused strategies and show greater sensitivity to functional disruptions, leading to stronger disability–mental health connections,^25^ whereas females’ tendency towards emotion-focused coping and social support may moderate disability impacts.^26^ Furthermore, societal expectations and gender norms may exacerbate these differences, with males with disabilities facing heightened pressure to conform to physical standards and participate in challenging activities.^20,27^

Our findings align with prior research documenting increased prevalence of mental health issues, particularly depression, during adolescence,^28–30^ while extending this through network analysis that reveals dynamic shifts in functional disability–mental well-being relationships across development. These changes likely stem from adolescent-specific factors such as hormonal changes, neurodevelopment and psychosocial stressors,^31–33^ alongside increasing educational and social barriers that may exacerbate disability-related challenges.^3,26,27,34^ These developmental changes appear more pronounced in females, potentially owing to earlier pubertal onset, more dramatic hormonal fluctuations affecting both physical and emotional functioning, heightened societal pressures regarding appearance and capabilities, and the development of gender-specific introspective coping strategies. However, longitudinal studies are needed to validate these proposed mechanisms.

Despite observed gender differences and developmental changes in mental well-being–functional disability networks, core disability domains – accepting change, making friends, behavioural control and remembering/concentrating – remain consistently influential across genders and developmental stages. These domains likely represent fundamental developmental tasks and coping mechanisms for children with functional disabilities.^35,36^ Accepting change and behavioural control facilitate management of challenges posed by disabilities,^37^ and making friends and remembering/concentrating are crucial for integration and academic achievement.^20,38,39^ This aligns with previous research identifying social and cognitive factors as key determinants of mental well-being in children with disabilities,^4,20,38,39^ while extending it by demonstrating their consistent significance across genders and identifying previously underemphasised factors such as accepting change and behavioural control.^37^

Our network analysis revealed stronger functional disability–mental well-being connections in males and evolving patterns across development. However, network analysis cannot establish causality or directionality. The stronger male associations could indicate either greater impact of disability on mental well-being, increased vulnerability to functional disabilities owing to mental health issues, or bidirectional relationships. Similarly, strengthening depression–disability connections in adolescence may reflect disabilities increasingly affecting mood, depressive symptoms heightening disability susceptibility, or complex bidirectional interactions. Longitudinal studies are necessary to determine temporal sequences and causal relationships across gender differences and developmental trajectories.

### Clinical implications

Our findings have important implications. The identification of consistent core disability domains across genders and developmental stages provides clear targets for intervention and support services. These findings enable tailored approaches: programmes for adolescent males should emphasise accepting change, whereas female-focused interventions should prioritise behavioural control, with social skills development (e.g. making friends) important for both genders.^20,37–39^ Males with functional disabilities warrant particular attention, given their stronger disability–mental health connections. Practitioners should consider developmental stages when designing interventions, with increased focus on depression screening during adolescence, particularly for individuals with difficulties in core domains (accepting change, social relationships, behavioural control and cognitive function).

### Strengths and limitations

Our study has strengths. The network analysis methodology provides novel insights into functional disability–mental well-being relationships while offering intuitive visualisation of these connections. The approach revealed key findings, including discrepancies between EI and BEI, suggesting that certain disabilities (e.g. learning and communication) may primarily affect disability-related symptoms rather than mental well-being directly. The study’s strengths also include its large sample size (*n* = 32 669), use of the validated CFM instrument enabling cross-country comparisons, and focus on underrepresented LMICs. The age- and gender-specific analyses illuminate developmental trajectories in disability–mental health relationships. Although BEI was our primary metric, future research could incorporate additional network parameters (e.g. clustering coefficient, characteristic path length) to further elucidate network structure and information flow. Additional network analyses of mental well-being in LMICs would enhance understanding of these relationships across diverse contexts.

Study limitations include methodological and measurement challenges. A primary constraint is the potential overlap between functional disability symptoms and mental health manifestations, particularly evident in cognitive and behavioural domains in which symptoms such as attention difficulties or impaired social interactions could indicate either condition. Although our cross-sectional design precludes causal inference, it identifies crucial markers for mental health concerns in young people with disabilities, particularly valuable for resource-limited settings. Second, the network analysis approach, although innovative, lacks traditional risk metrics (odds ratios and/or risk ratios) with benchmark value and assumes symmetric bidirectional relationships, which may not reflect reality. Mental well-being assessment was limited to two caregiver-reported items, potentially missing broader manifestations of depression/anxiety (irritability, sleep disorders, fatigue, anhedonia). The CFM’s reliance on proxy reporting introduces inherent limitations regarding awareness of ‘internalising’ problems, although reported prevalence aligns with global estimates and the instrument has demonstrated robust psychometric properties through extensive testing.^4^ Important unmeasured confounders include maltreatment, bullying, stigma and parental mental health.

## Supporting information

Chen et al. supplementary material Chen et al. supplementary material

## Data Availability

The data used in our analysis are publicly available and can be accessed at https://mics.unicef.org/surveys.

## Supplementary material

The supplementary material is available online at https://doi.org/10.1192/bjp.2024.278.
