# Supplementary material for: Network analysis of functional disabilities and their association with mental well-being in children and adolescents: multi-country study across low- and middle-income countries
Source: Br J Psychiatry. 2025 Mar 19;228(2):116–25. doi: 10.1192/bjp.2024.278 (PMC12823452; doi:10.1192/bjp.2024.278)

**Title: Network analysis of functional disabilities and their association with mental well-being in children and adolescents: a multi-country study across 47 countries**

**Supplementary Figure 1. Flowchart of the samples in the Multiple Indicator Cluster Surveys.**


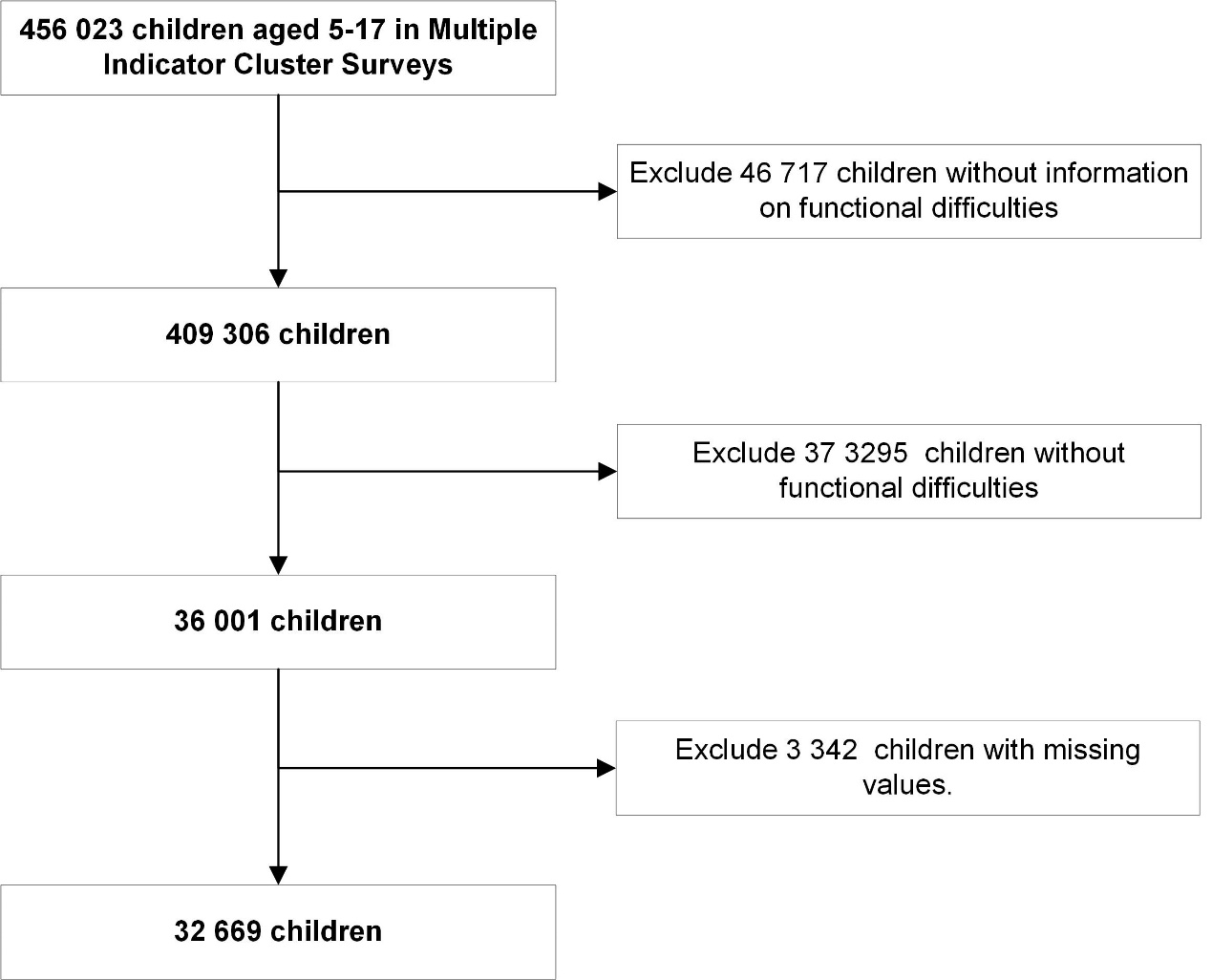


**Supplementary Figure 2. Expected influence of nodes for functional disability and mental well-being, by sex.** A higher expected influence signifies that a particular node has a stronger association with all other nodes in the network. P values, comparing the two conditions for each node, were extracted from the permutation test. (Sorted by expected influence for males.)


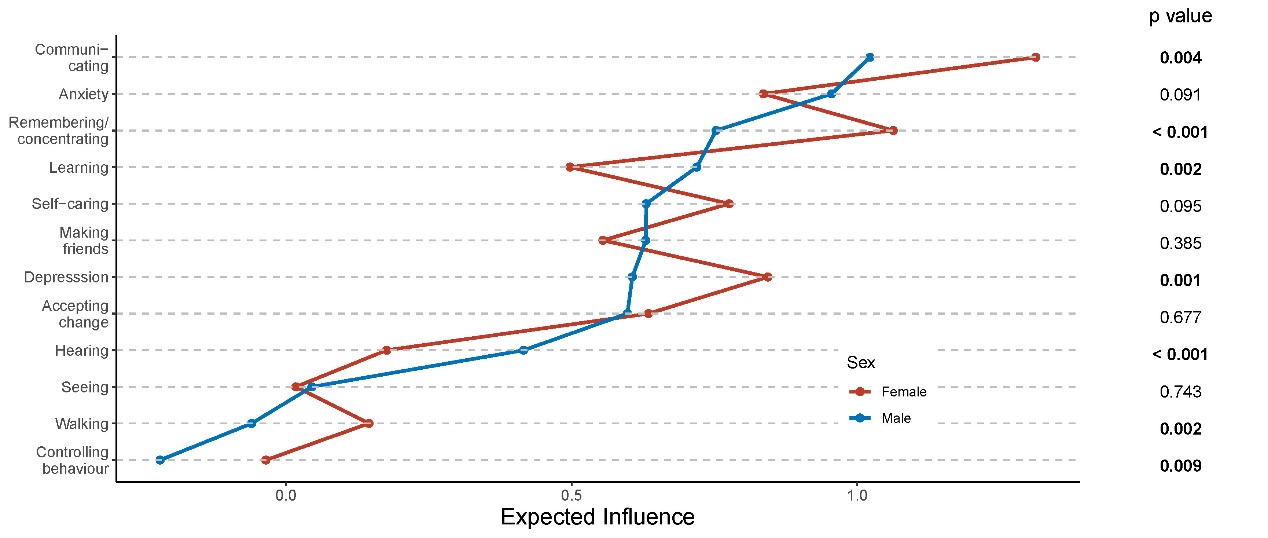


**Supplementary Figure 3. Expected influence of nodes for functional disability and mental well-being among females, by age.** Conventions as for Supplementary Figure 2. (Sorted by expected influence for adolescents.)


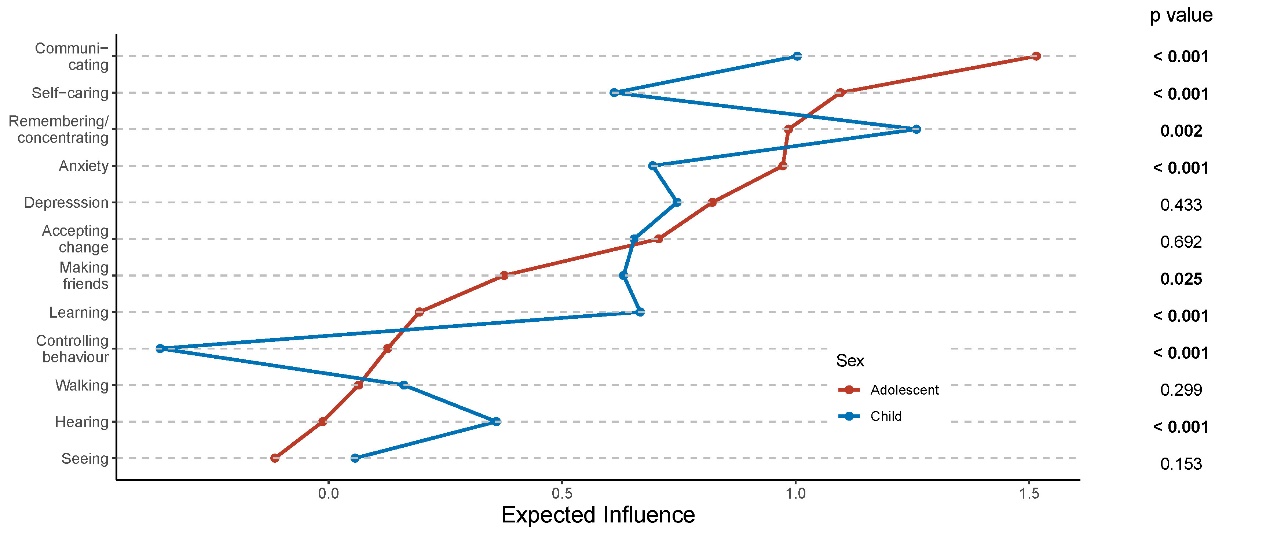


**Supplementary Figure 4. Expected influence of nodes for functional disability and mental well-being among males, by age.** Conventions as for Supplementary Figures 2/3.


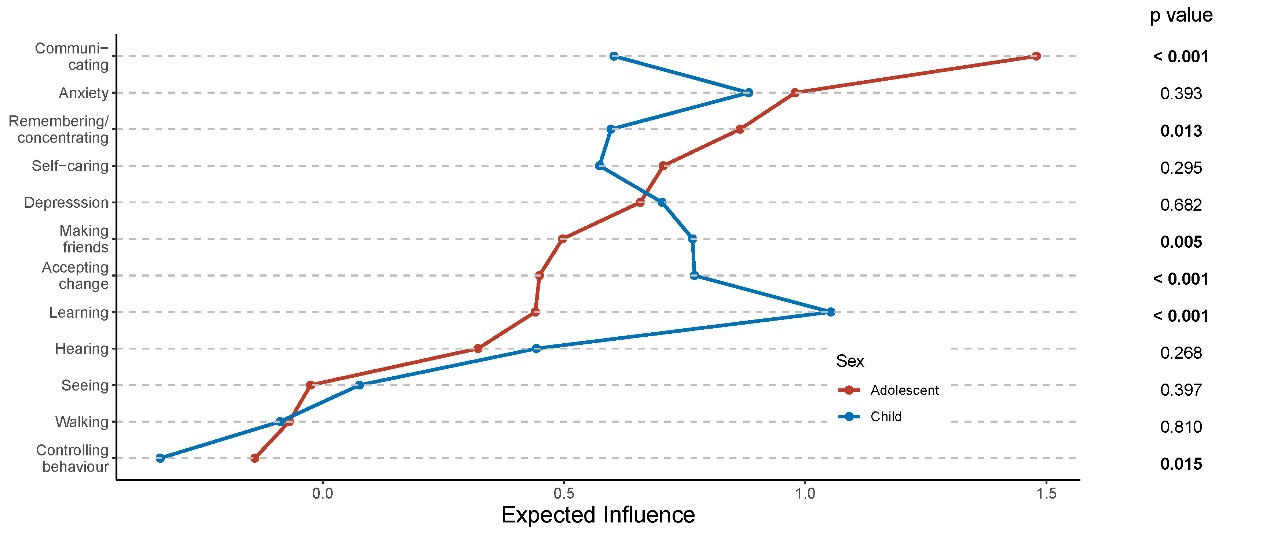


**Supplementary Figure 5. Network stability among females tested by a nonparametric bootstrapping method.** Red points show the corresponding values based on original samples. Black points and grey area show the corresponding values and their confidence intervals (CIs) based on the bootstrapped samples. Less overlapping of CIs suggests greater accuracy of edge or centrality.


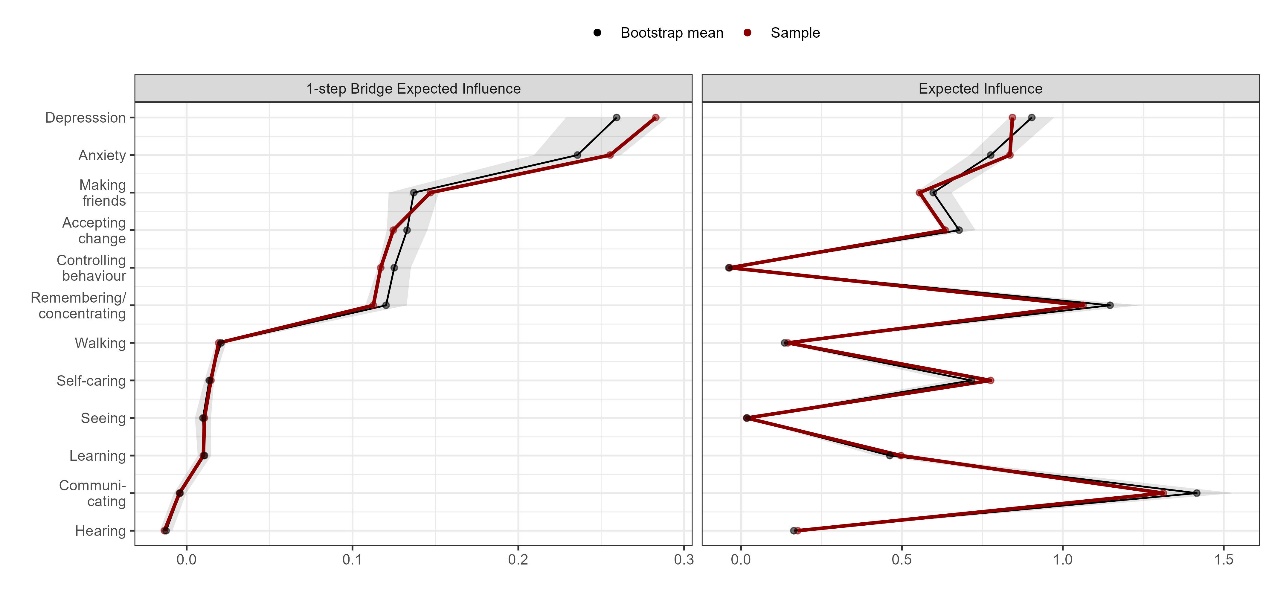


**Supplementary Figure 6. Network stability among females aged 5–9 tested by nonparametric bootstrapping method.** Conventions as for Supplementary Figure 5.


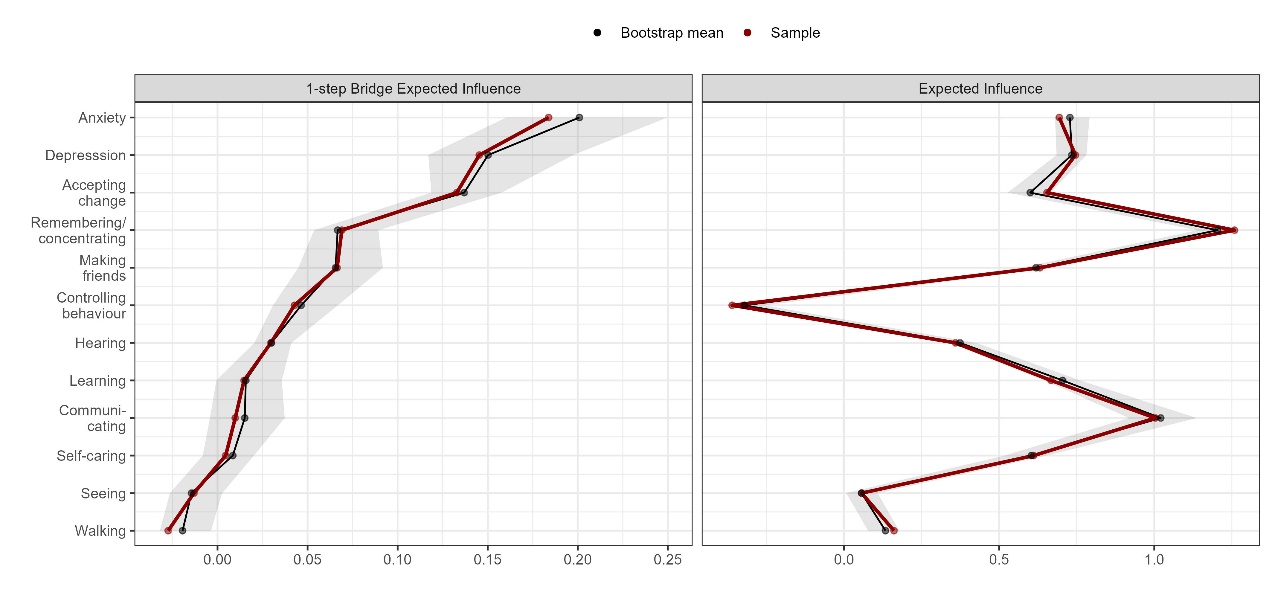


**Supplementary Figure 7. Network stability among females aged 10–17 tested by a nonparametric bootstrapping method.** Conventions as for Supplementary Figure 5.


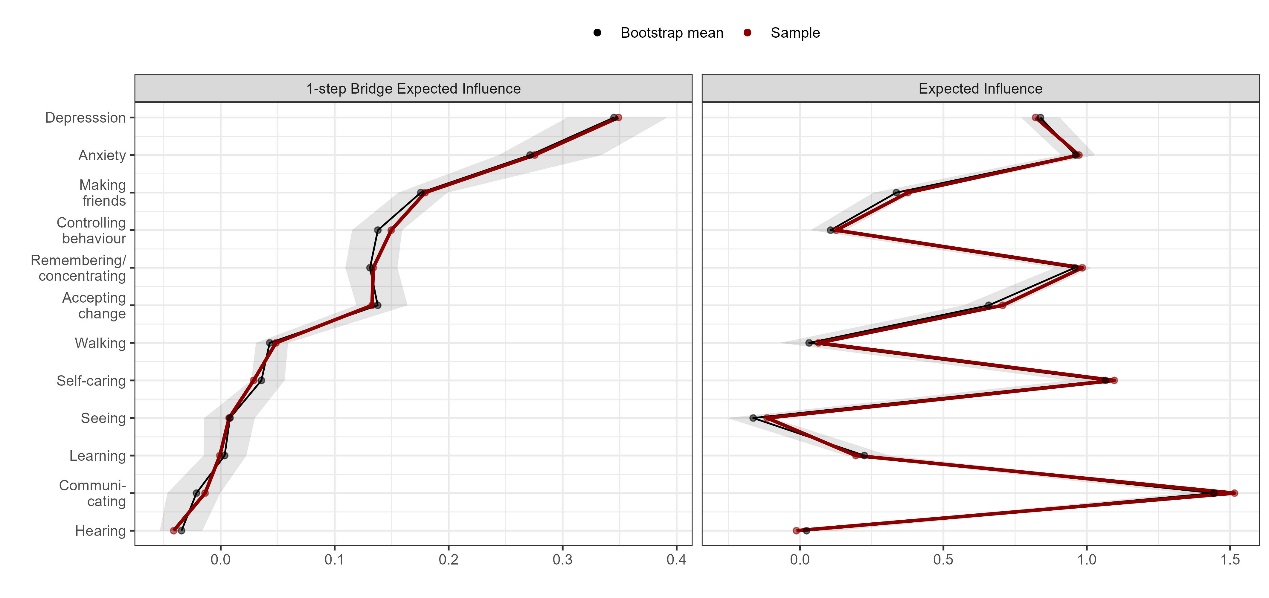


**Supplementary Figure 8. Network stability among males tested by a nonparametric bootstrapping method.** Conventions as for Supplementary Figure 5.


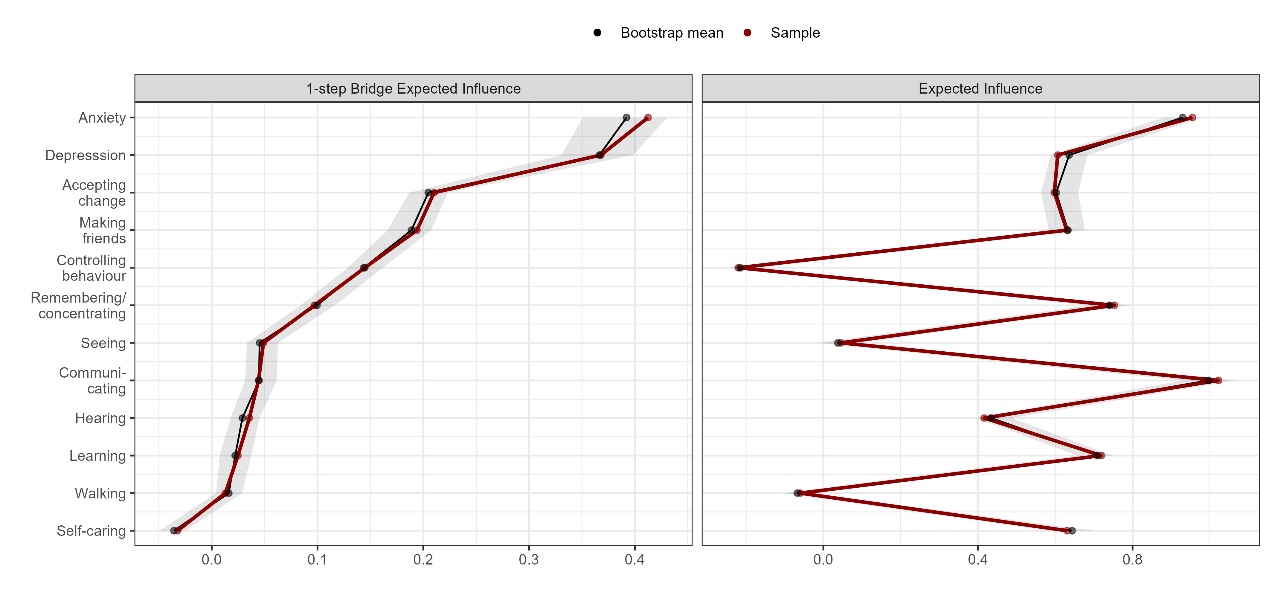


**Supplementary Figure 9. Network stability among males aged 5–9 tested by a nonparametric bootstrapping method.** Conventions as for Supplementary Figure 5.
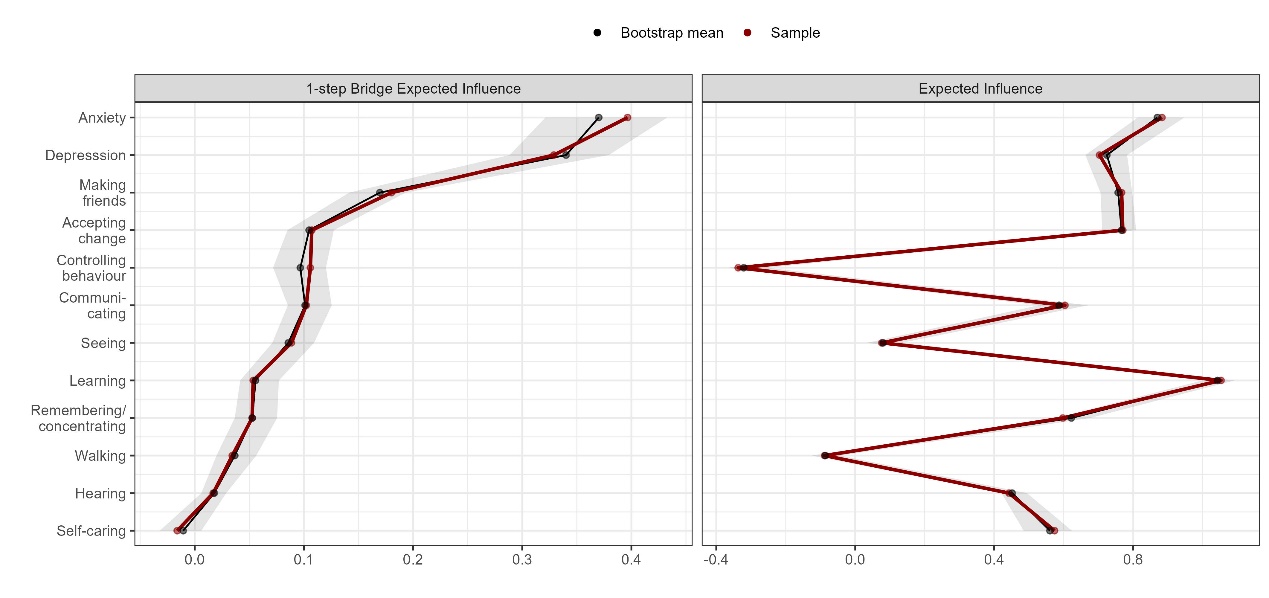


**Supplementary Figure 10. Network stability among males aged 10–17 tested by a nonparametric bootstrapping method.** Conventions as for Supplementary Figure 5.


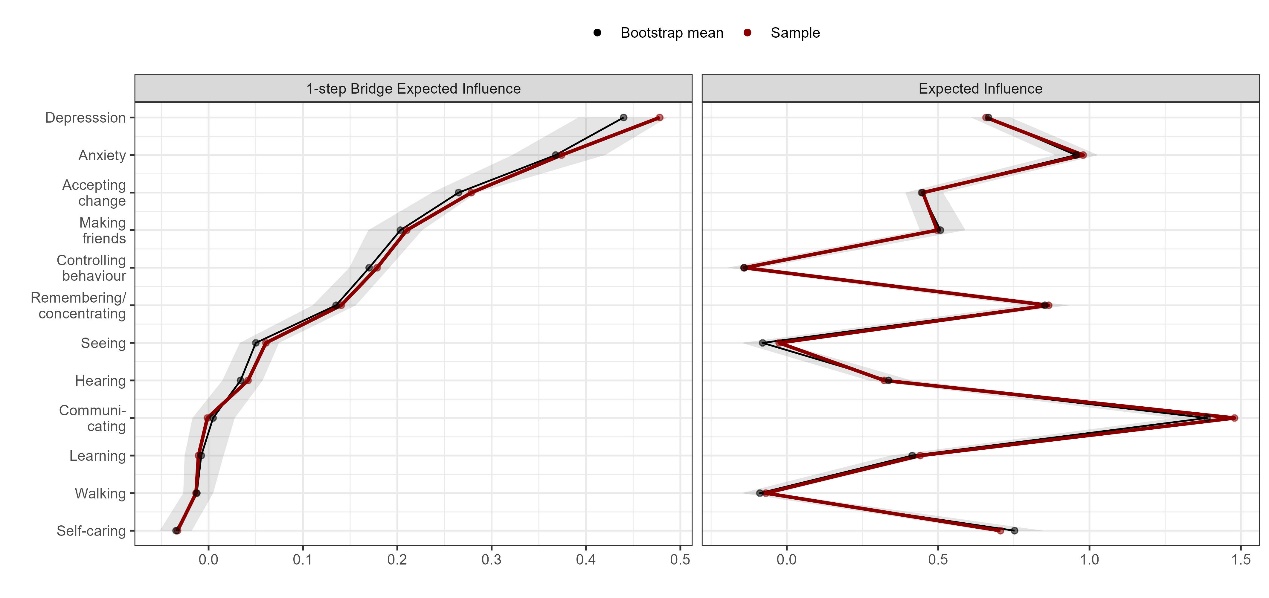


**Supplementary Figure 11. Network stability among females tested by a case-dropping bootstrap method.** The *x* axis represents the percentage of cases of the original included participants that remained at each case-dropping subset. The *y* axis represents the average of correlations between the centrality indexes from the original network and the re-estimated network after the case-dropping procedure.


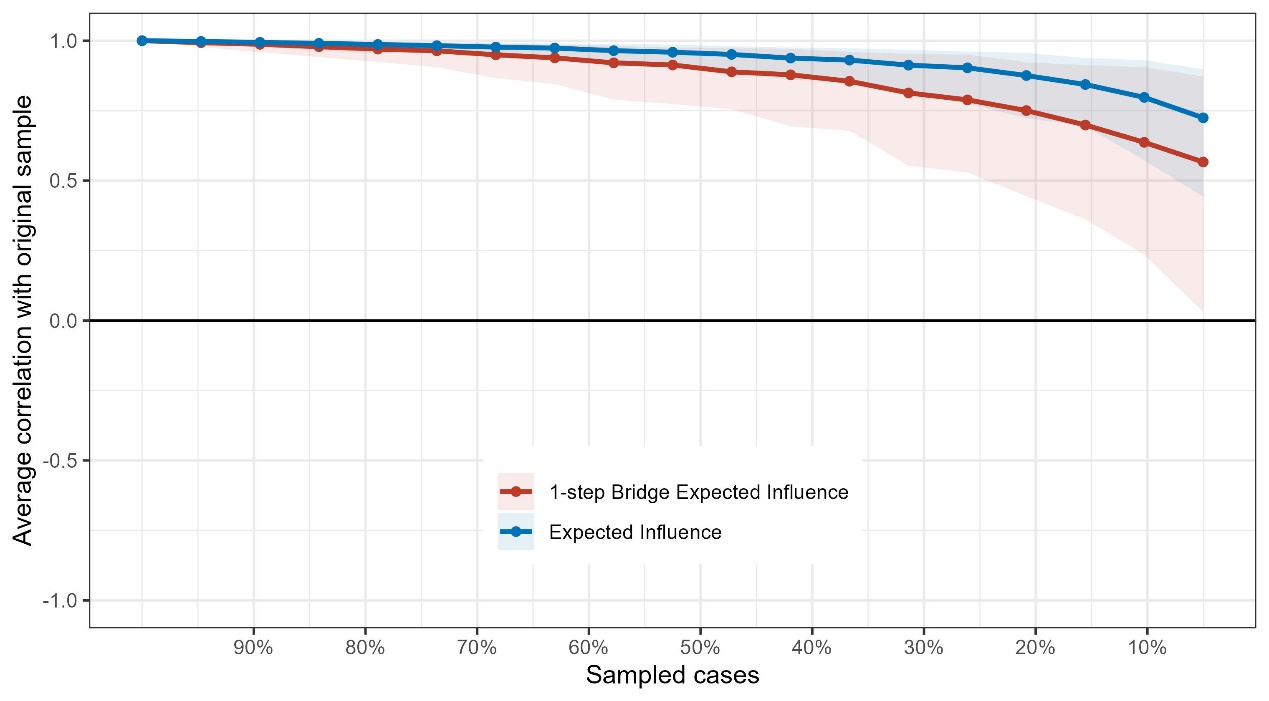


**Supplementary Figure 12. Network stability among females aged 5–9 tested by a case-dropping bootstrap method.** Conventions as for Supplementary Figure 11.
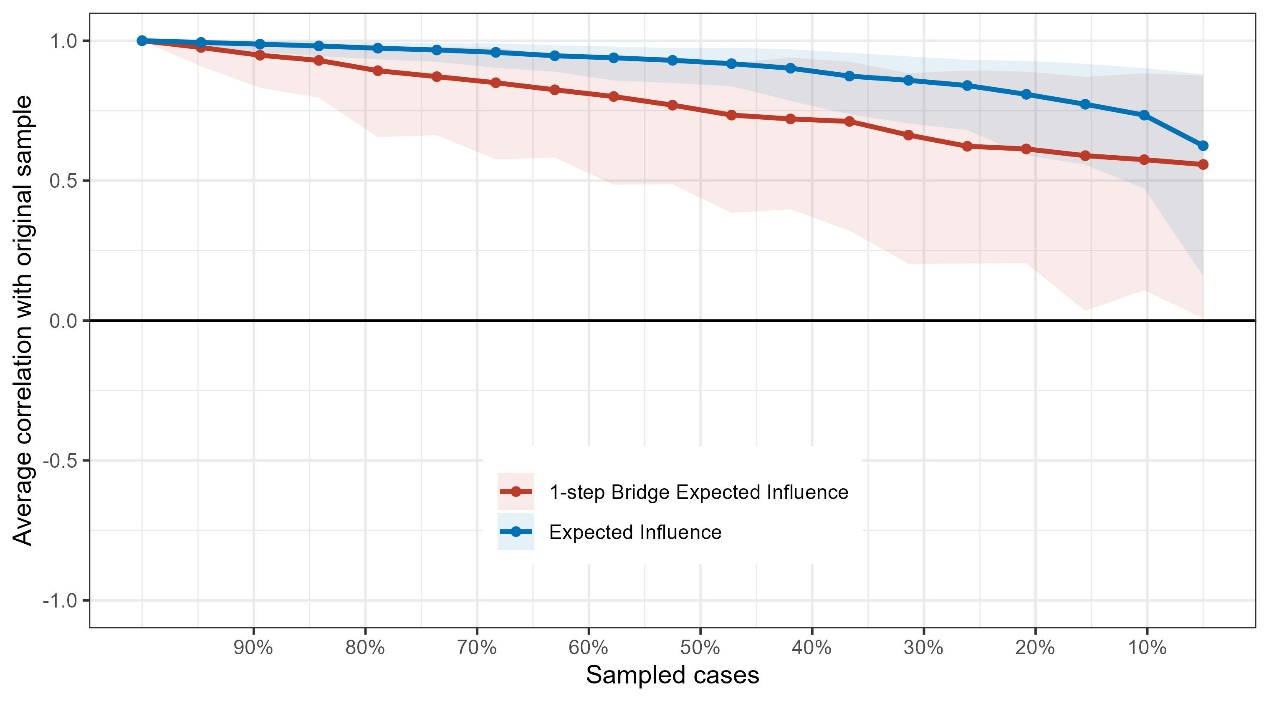


**Supplementary Figure 13. Network stability among females aged 10–17 tested by a case-dropping bootstrap method.** Conventions as for Supplementary Figure 11.


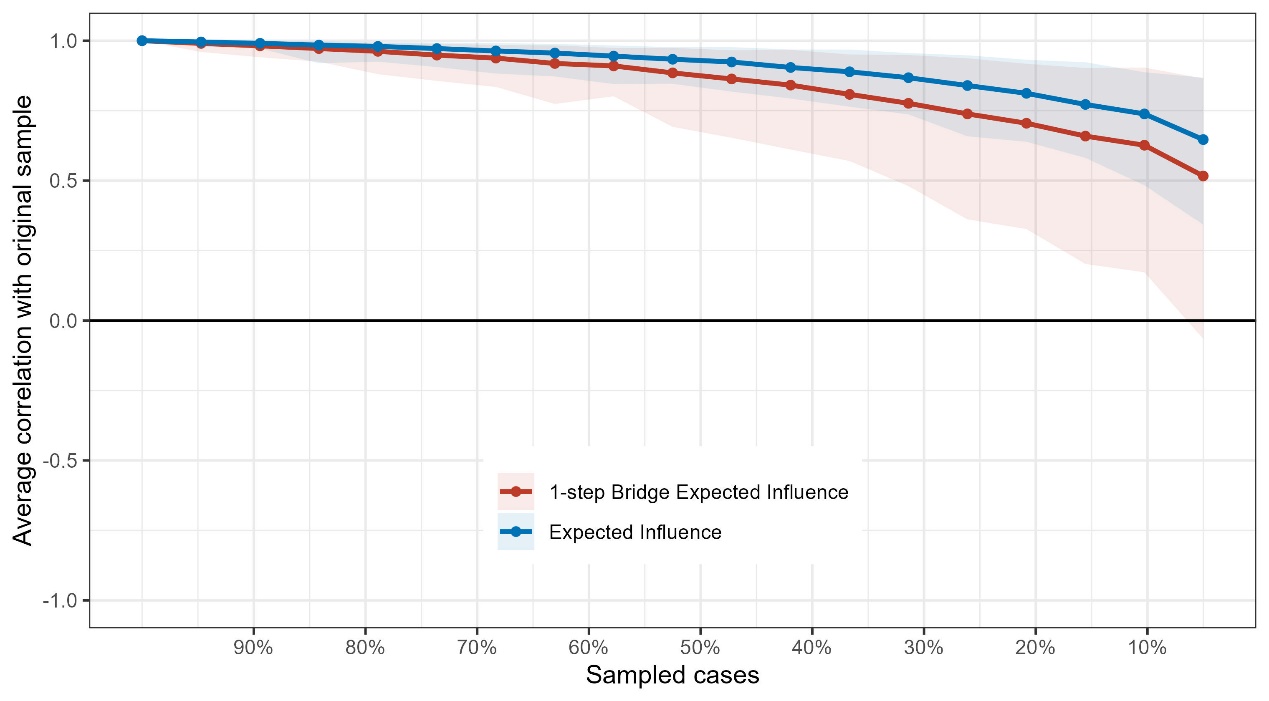


**Supplementary Figure 14. Network stability among males tested by a case-dropping bootstrap method.** Conventions as for Supplementary Figure 11.


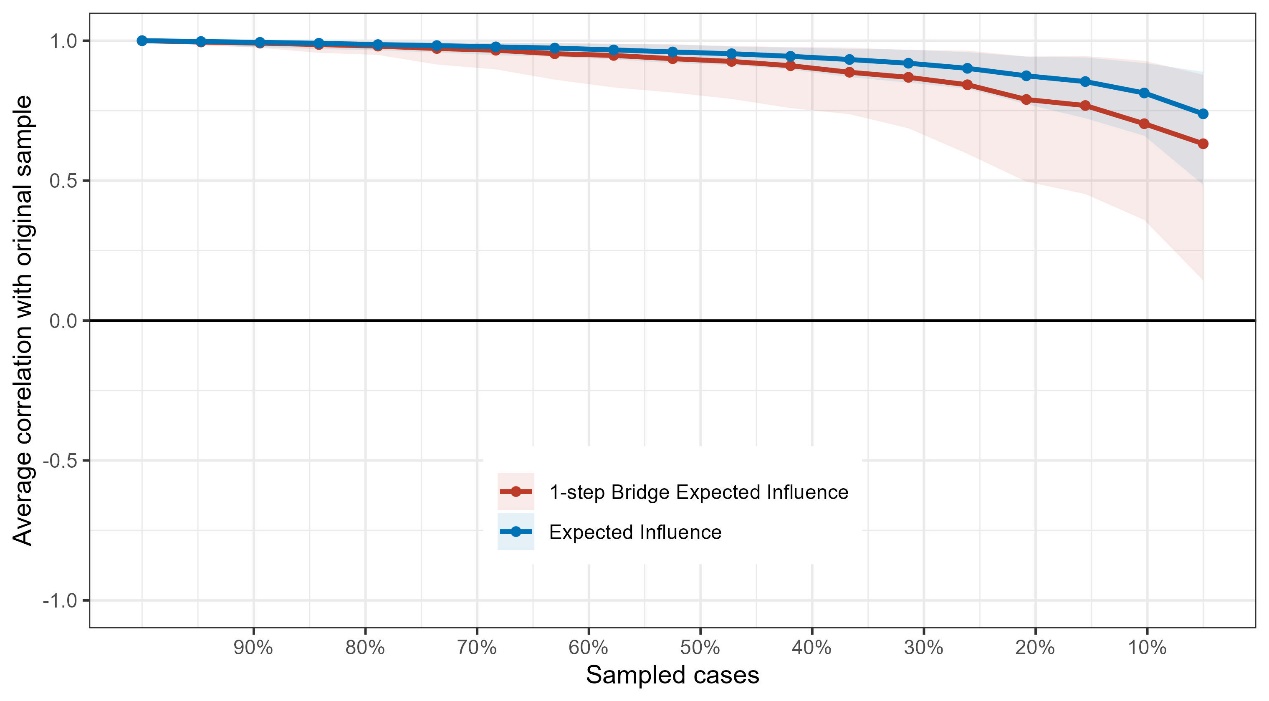


**Supplementary Figure 15. Network stability among males aged 5–9 tested by a case-dropping bootstrap method.** Conventions as for Supplementary Figure 11.


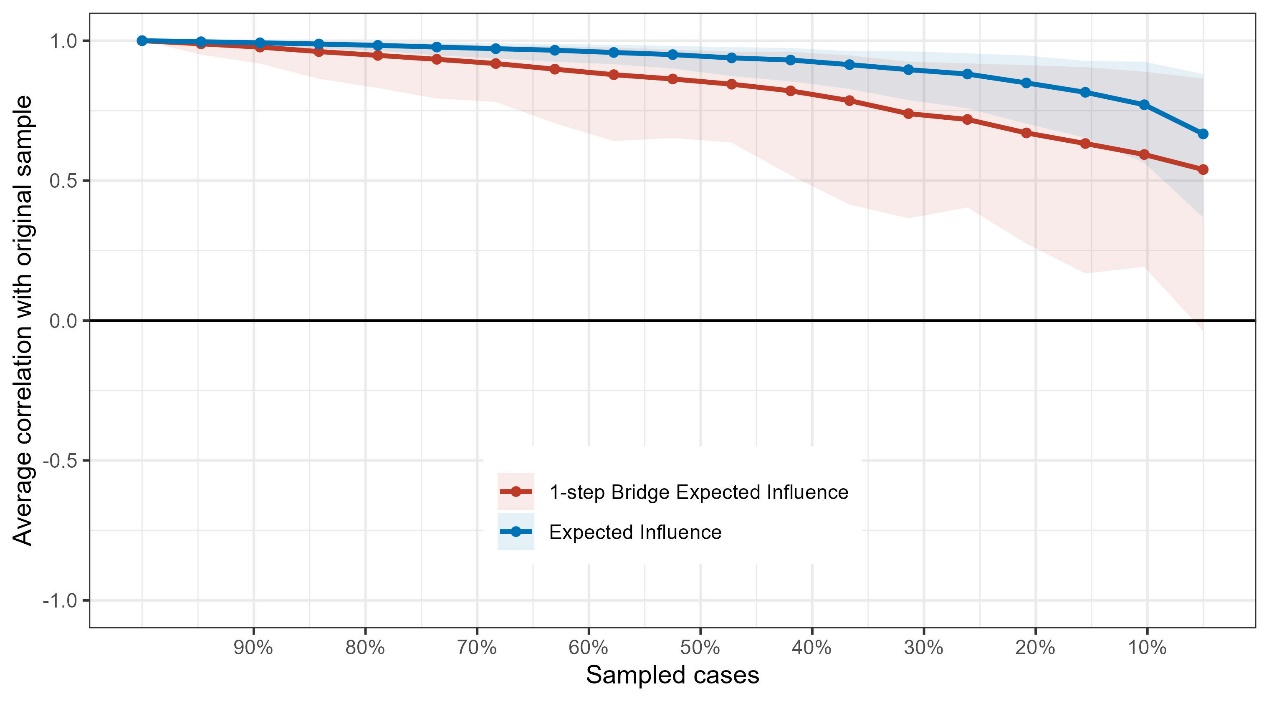


**Supplementary Figure 16. Network stability among males aged 10–17 tested by case-dropping bootstrap method.** Conventions as for Supplementary Figure 11.


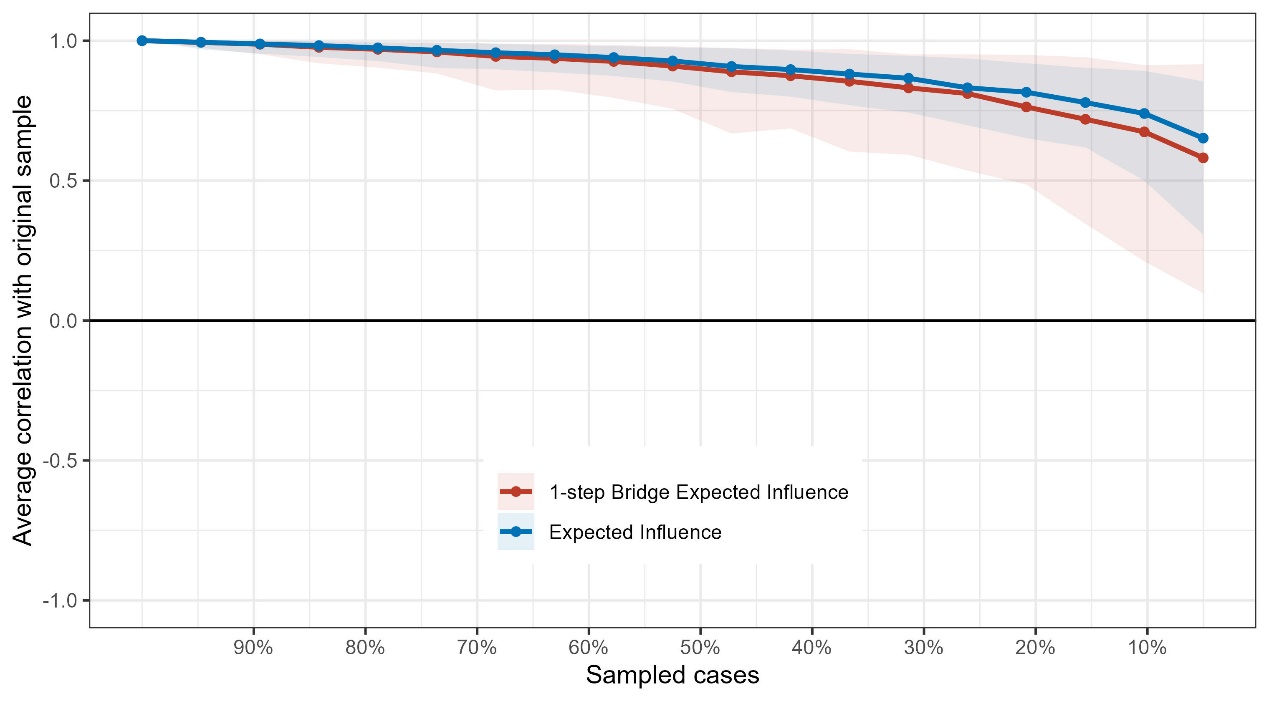


**Supplementary Figure 17. Network structure between functional disability and mental well-being, by sex,** **after imputation of missing data.** The nodes with different colours represent the “communities” of mental well-being, disabilities, and confounders. Edges represent the connections or associations between nodes, with thicker edges indicating stronger associations. A solid edge means a positive association, and a dashed edge means a negative association. For mental well-being, “depression”/“anxiety” means more depression or anxiety. For functional disability, “seeing”, for example, means difficulties in seeing.


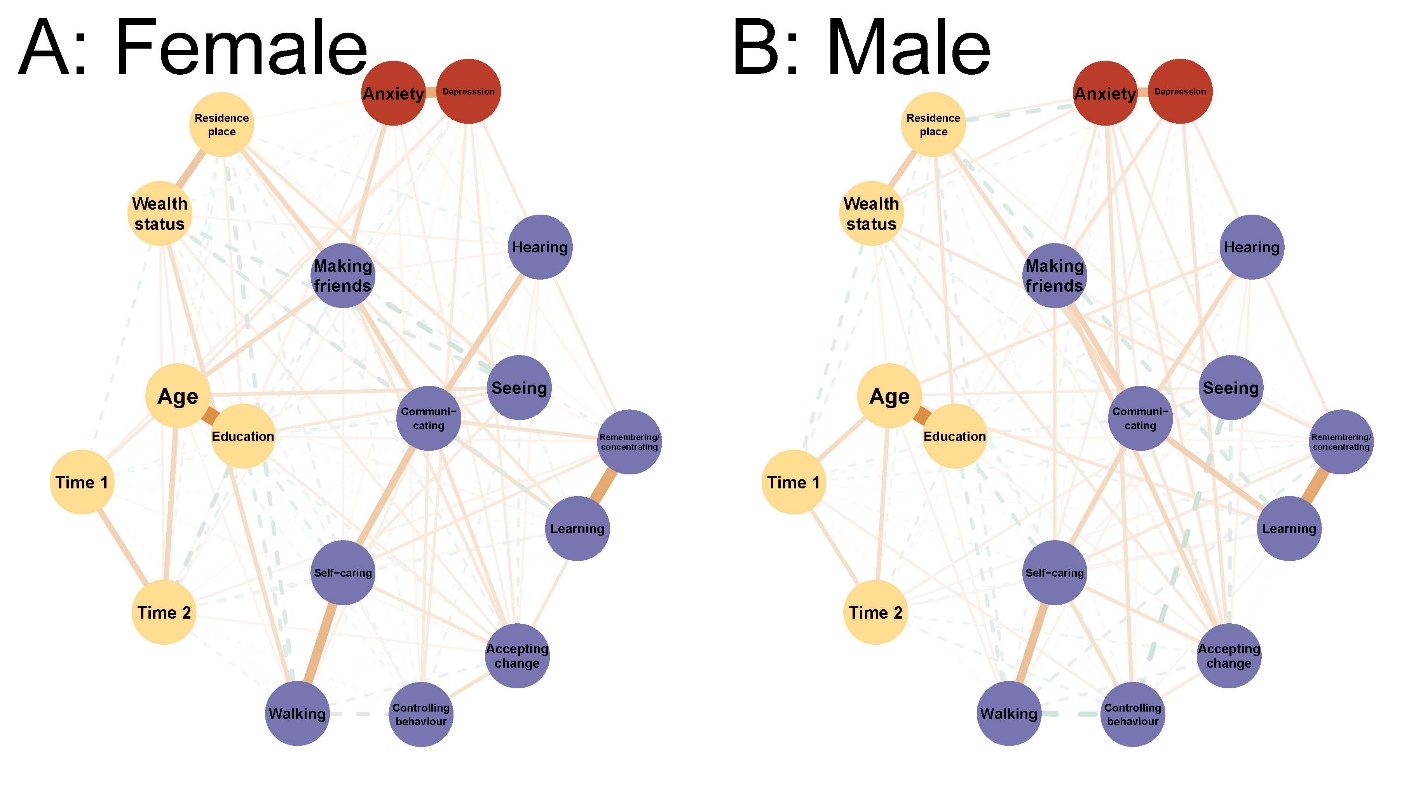


**Supplementary Figure 18**. **Bridge expected influence of nodes for functional disability and mental well-being, by sex, after imputation of missing data**. A node with a higher bridge expected influence serves as a more important link between the “community” of functional disability nodes and the community of mental well-being nodes. *P* values, comparing the two conditions for each node, were extracted from the permutation test and corrected by Benjamini-Hochberg method. For mental well-being, “depression”/“anxiety” means more depression/anxiety. For functional disability, “seeing”, for example, means difficulties in seeing.


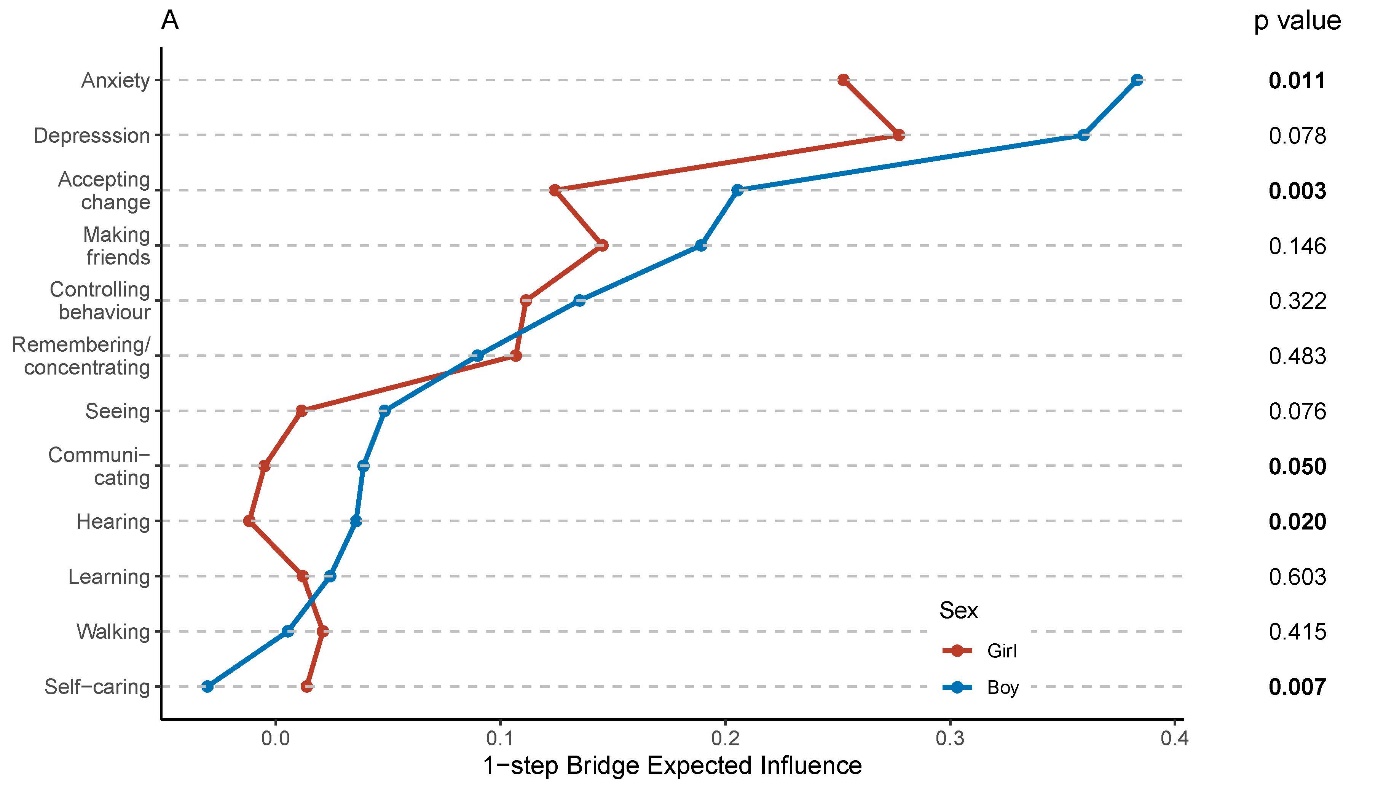


**Supplementary Figure 19. Network structure between functional disability and mental well-being among females, by age, after imputation of missing data.** Graphical conventions as for Supplementary Figure 17.


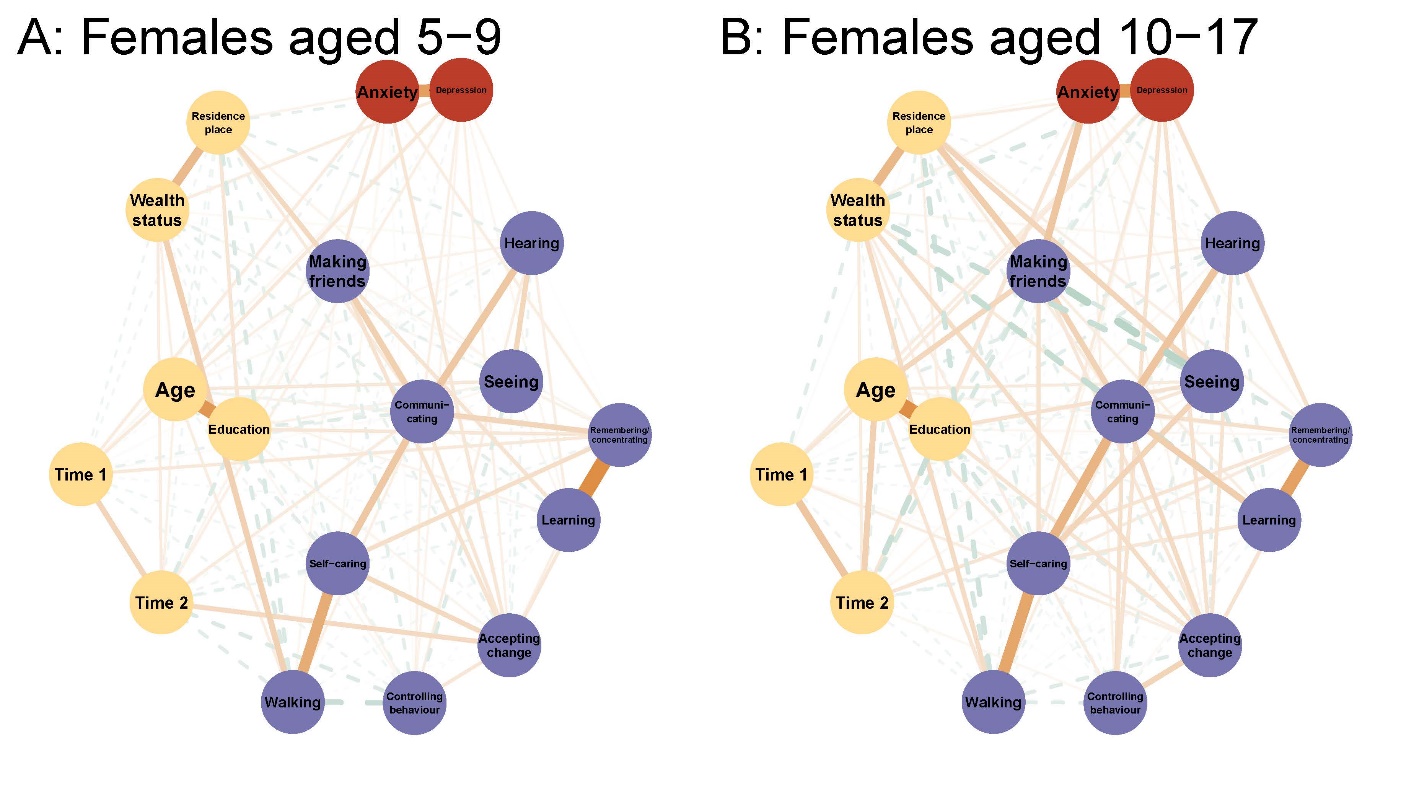


**Supplementary Figure 20. Network structure between functional disability and mental well-being among males, by age, after imputation of missing data.** Graphical conventions as for Supplementary Figure 17.


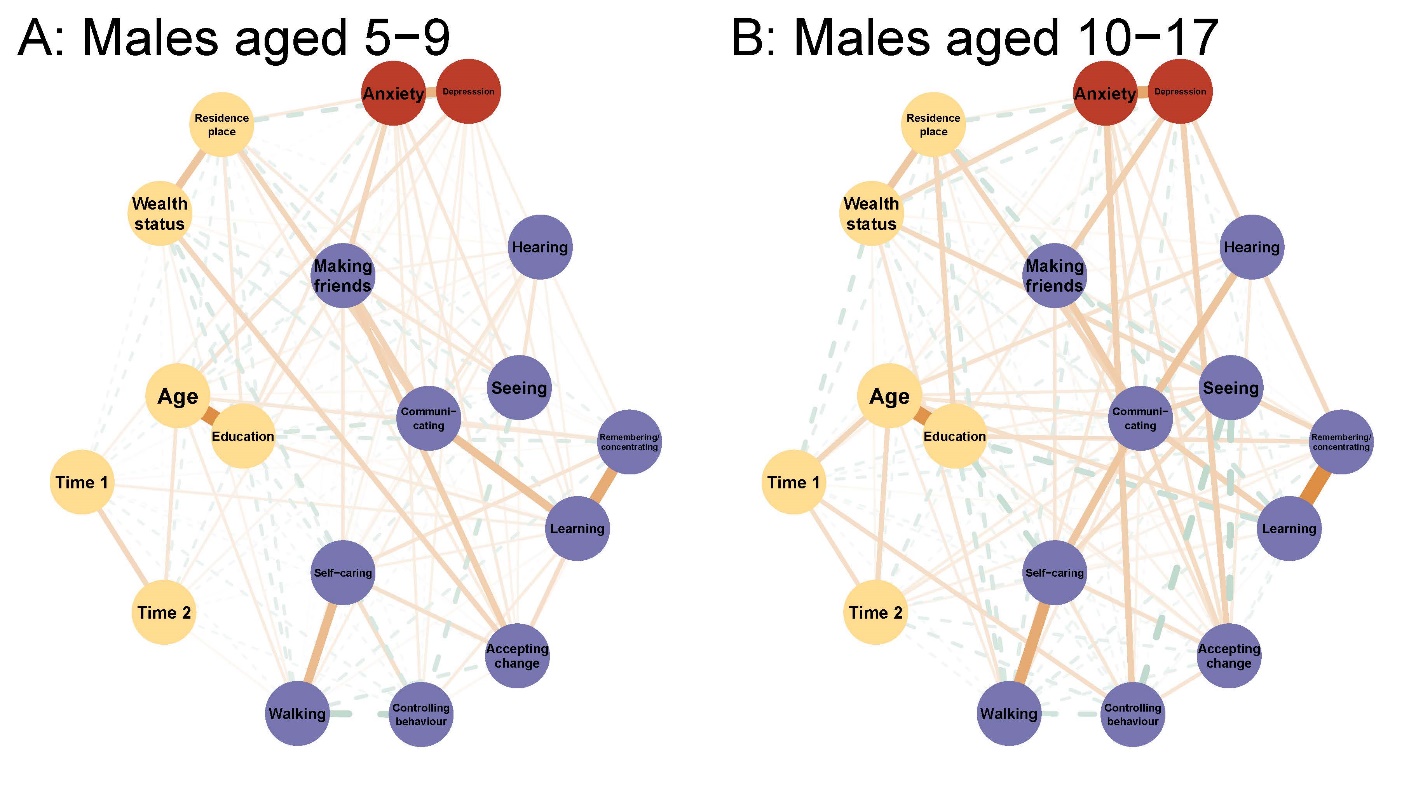


**Supplementary Figure 21.** **Bridge expected influence of nodes for functional disability and mental well-being, by sex and by age, after imputation of missing data**. Conventions as for Supplementary Figure 18. Factors sorted within each sex by the bridge expected influence for adolescents.


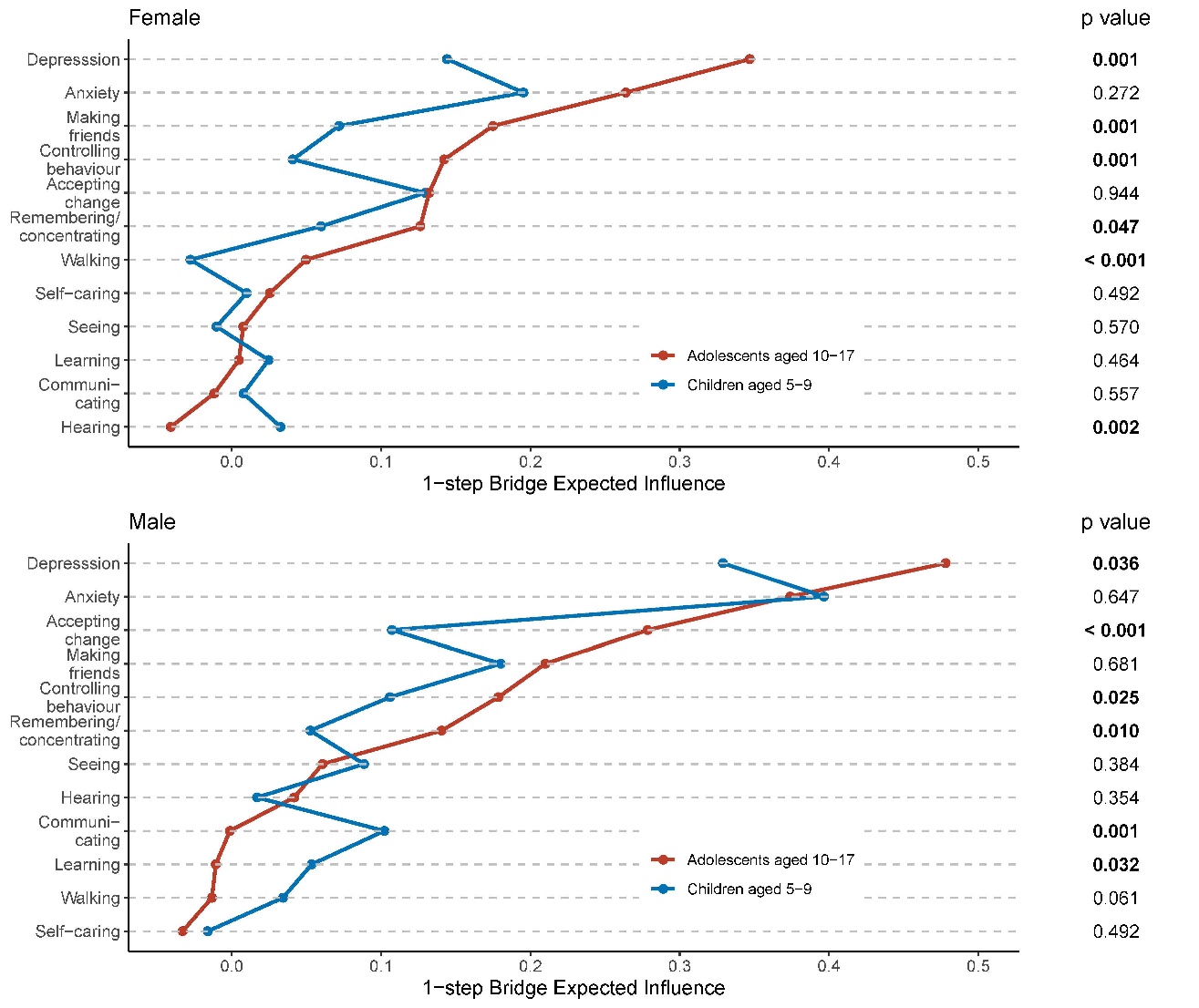

Supplement: Chen et al. supplementary material [file S0007125024002782sup001.docx]
